# The ‘Ventral Organs’ of Pycnogonida (Arthropoda) Are Neurogenic Niches of Late Embryonic and Post-Embryonic Nervous System Development

**DOI:** 10.1371/journal.pone.0095435

**Published:** 2014-04-15

**Authors:** Georg Brenneis, Gerhard Scholtz

**Affiliations:** Humboldt-Universität zu Berlin, Institut für Biologie/Vergleichende Zoologie, Berlin, Germany; University of Pécs Medical School, Hungary

## Abstract

Early neurogenesis in arthropods has been in the focus of numerous studies, its cellular basis, spatio-temporal dynamics and underlying genetic network being by now comparably well characterized for representatives of chelicerates, myriapods, hexapods and crustaceans. By contrast, neurogenesis during late embryonic and/or post-embryonic development has received less attention, especially in myriapods and chelicerates. Here, we apply *(i)* immunolabeling, *(ii)* histology and *(iii)* scanning electron microscopy to study post-embryonic ventral nerve cord development in *Pseudopallene* sp., a representative of the sea spiders (Pycnogonida), the presumable sister group of the remaining chelicerates. During early post-embryonic development, large neural stem cells give rise to additional ganglion cell material in segmentally paired invaginations in the ventral ectoderm. These ectodermal cell regions – traditionally designated as ‘ventral organs’ – detach from the surface into the interior and persist as apical cell clusters on the ventral ganglion side. Each cluster is a post-embryonic neurogenic niche that features a tiny central cavity and initially still houses larger neural stem cells. The cluster stays connected to the underlying ganglionic somata cortex via an anterior and a posterior cell stream. Cell proliferation remains restricted to the cluster and streams, and migration of newly produced cells along the streams seems to account for increasing ganglion cell numbers in the cortex. The pycnogonid cluster-stream-systems show striking similarities to the life-long neurogenic system of decapod crustaceans, and due to their close vicinity to glomerulus-like neuropils, we consider their possible involvement in post-embryonic (perhaps even adult) replenishment of olfactory neurons – as in decapods. An instance of a potentially similar post-embryonic/adult neurogenic system in the arthropod outgroup Onychophora is discussed. Additionally, we document two transient posterior ganglia in the ventral nerve cord of *Pseudopallene* sp. and evaluate this finding in light of the often discussed reduction of a segmented ‘opisthosoma’ during pycnogonid evolution.

## Introduction

In the most diverse animal lineage, the Arthropoda, our understanding of the early neurogenic processes that lie at the base of central nervous system formation is founded on numerous studies over several decades. Among the four major arthropod groups – Chelicerata, Myriapoda, Hexapoda and the most likely paraphyletic crustaceans [1]–[6] – early neurogenesis is best investigated in hexapods. Especially for the well established laboratory organisms *Drosophila melanogaster*, *Tribolium castaneum*, and different grasshopper species (*Schistocerca* sp. and *Locusta* sp.) we have extensive knowledge of the involved neural precursor cell types [7]–[13], the origin of neural cell lineages and their contribution to adult structures [12], [14]–[18], and the underlying genetic network [13], [19]–[25]. However, also in the other groups considerable new insights into early neurogenesis have been gained during the last 25 years (Chelicerata: [26]–[32]; Myriapoda: [32]–[37]; crustaceans: [38]–[49]). Remarkably, several striking similarities and differences have been revealed between the neurogenic mechanisms in the different arthropod lineages, providing compelling arguments to the debate on their phylogenetic relationships [47], [48], [50]–[53].

In contrast to the early embryonic phase, neurogenesis during late embryonic development and in post-embryonic stages (in case of indirectly developing arthropods) has received significantly less ‘arthropod-wide’ attention. Outside of hexapods [7], [54]–[57], the cellular basis and dynamics of late neurogenesis in the further developed and often already functional central nervous system (CNS) has been addressed only in few studies on malacostracan crustaceans [42], [58]–[61], even more rarely in chelicerates [62], [63] and not been followed at all in recent myriapod investigations. Likewise, the (potential) occurrence of adult neurogenesis in so-termed neurogenic niches, i.e., cellular microenvironments that produce neural cell material during the entire life-span of an organism, has been investigated only in decapod crustaceans [64]–[69], and to a lesser extent in some hexapod representatives [70]–[75]. Apart from some histological studies, compelling data on myriapods and chelicerates are lacking [75]–[77]. Therefore, present-day studies on late nervous system development in myriapods and chelicerates with a focus at the cellular level are highly desirable and represent a prerequisite to enable arthropod-wide comparison of neurogenic mechanisms across the entire development [53]. Such investigations promise to provide additional arguments in the discussion on the phylogenetic relationships of arthropods and eventually help to unravel the evolution of neurogenic processes within this diverse animal group.

To contribute to this intriguing field, we studied post-embryonic nervous system development in *Pseudopallene* sp. (Pycnogonida, Callipallenidae), a representative of the exclusively marine sea spiders. The majority of recent phylogenetic analyses recover these spindly-legged arthropods as sister group of the remaining chelicerates [2], [3], [78], [79]. Owing to this position, pycnogonids are thought to have retained features that are plesiomorphic for chelicerates – if not even for arthropods as a whole. In previous studies, we have already described general embryonic and post-embryonic development of *Pseudopallene* sp. [80], [81], as well as embryonic neurogenesis of the ventral nerve cord (VNC) [53]. We have shown that paired apical invaginations form in each ventral neuromere during advanced embryonic development of *Pseudopallene* sp. These invaginations are lined by large neural stem cells (NSCs), which show high proliferation activity and give rise to future ganglion cell material via asymmetrical divisions. The apical ectodermal cell regions that comprise the invaginations with the NSCs are confluent with the underlying segmental ganglion anlagen and have already been mentioned in older pycnogonid studies [82]–[87], where they have been termed ‘ventral organs’ [82]. Yet, the exact fate of these CNS-associated ‘ventral organs’ and their function during subsequent post-embryonic development have remained disputed and rather elusive.

Here, we combine *(i)* fluorescent histochemical stainings and immunolabeling with confocal laser-scanning microscopy and subsequent computer-aided analysis, *(ii)* classical histology and *(iii)* scanning electron microscopy to follow post-embryonic nervous system development in *Pseudopallene* sp. from the hatching stage up to the adult. We show that the segmentally paired invaginations (‘ventral organs’) formed during embryogenesis continue to deepen and eventually detach from the apical surface of the ventral ectoderm. The internally detached cell regions persist as paired clusters that initially still house larger NSCs, representing post-embryonic neurogenic niches. Each cluster stays connected to the underlying ganglionic somata cortex via an anterior and a posterior cell stream, thus forming what we call a cluster-stream-system (CSS). We demonstrate a continuing increase of ganglion cell numbers due to the proliferation activity of cells in the CSSs and point out striking similarities to the well characterized adult neurogenic systems in the brain of decapod crustaceans. Additionally, we document the formation of two transient posterior ganglion anlagen during post-embryonic development of *Pseudopallene* sp. This finding is discussed in light of the frequently assumed reduction of a segmented ‘opisthosoma’ during pycnogonid evolution.

## Material and Methods

### Terminology

Distinction of post-embryonic stages (PSs) of *Pseudopallene* sp. follows Brenneis et al. [81].

For descriptions of nervous system development, the terminology suggested by Richter et al. [88] has been followed whenever applicable. As in our previous study on embryonic neurogenesis [53], the term ‘neurogenesis’ is used in a restrictive manner, covering only the processes that lead to the production of post-mitotic but still immature neurons and glial cells. Hence, all subsequent differentiation processes of post-mitotic neural cells are here excluded from neurogenesis. The term ‘neural precursor’ (NP) designates a progenitor cell that has already entered the neural pathway but is not yet post-mitotic. The neutral term ‘ganglion cell’ (GC) has been chosen for post-mitotic neurons and glial cells in all differentiation stages, unequivocal distinction especially between immature cells of both types being not possible with the used techniques.

At the species level, all pycnogonid names were updated to the current suggestions by Bamber and El Nagar [89].

A list of all abbreviations used in the text and in the figures is given in Table 1.

**Table 1:** List of abbreviations used in the text and figure legends.

ad	- adult	NP	- neural precursor
apc	- apical cell cluster	NSC	- neural stem cell
cec	- circum-esophageal connective	ov	- ovigeral neuromere
ch	- chelifore	pa	- palpal neuromere
CNS	- central nervous system	pg	- posterior ganglion anlage
con	- connective	PH3	- phospho-histone H3
CSS	- cluster-stream-system	pn	- proctodeal nerve
EC	- ectodermal cell	PS	- post-embryonic stage
EPC	- epidermis cell	pvc	- postero-ventral commissure
eso	- esophagus	seg	- sub-esophageal ganglion
GC	- ganglion cell	sub-ad	- sub-adult
GN	- glomerulus-like neuropil	VNC	- ventral nerve cord
INP	- intermediate neural precursor	wl	- walking leg (segment)
mg	- midgut	wlg	- walking leg ganglion (anlage)
mgd	- midgut diverticulum		

### Specimen collection and fixation

Details on the collection of the different PSs of *Pseudopallene* sp. are given in Brenneis et al. [81]. Fixation of developmental stages was carried out at ambient temperature. For all fluorescence stainings and scanning electron microscopy, specimens were fixed in PFA/SW (16% formaldehyde in ddH_2_0 (methanol-free, Electron Microscopy Sciences, #15710) diluted 1∶4 in filtered natural sea water). Fixation was conducted either for 30–40 min with subsequent gradual transfer into absolute methanol for long-term storage, or over a prolonged time span (several days at ambient temperature plus some weeks at 4°C) with subsequent transfer into phosphate buffered saline (PBS,1.86 mM NaH_2_PO_4_, 8.41 mM Na_2_HPO_4_, 17.5 mM NaCl; pH 7.4) containing 0.1% NaN_3_. For histology, embryos were fixed in Bouin's solution (15 parts saturated aqueous picric acid, 5 parts 37% formaldehyde (methanol-stabilized), 1 part glacial acetic acid) for 30–40 min, followed by repeated thorough washing and long-term storage in 70% ethanol.

### Specimen dissection and fluorescent staining procedures

For most fluorescent stainings, the complete CNS was dissected using ground dissection needles or electrochemically etched tungsten tips in combination with sharpened watchmaker forceps (Dumont 5). For immunohistochemistry, samples were initially washed in several changes of PBTx (0.3% Triton X-100, 0.5% bovine serum albumine, 1.5% DMSO (dimethylsulfoxide) in PBS) for ≥2 h and then blocked for ≥1 h in PBTx+N (5% Normal Goat Serum (Dako, #X0907) in PBTx) prior to antibody exposure. Primary and secondary antibodies (see below) were diluted in PBTx+N, incubation times lasted at minimum overnight, being sometimes extended up to 72 h. Each antibody incubation was followed by washing in PBTx on a horizontal shaker (NeoLab DOS-20S, 55–70 rpm) for at least 4 h at RT with several changes of the buffer. Omission of primary antibodies resulted in complete signal loss. Nuclear counterstaining was performed with Hoechst (H33342, Invitrogen Molecular Probes, #H1399, 1 µg/ml in PBS) following the preceding labeling procedures. Hoechst incubation lasted at least 1 h and was occasionally extended overnight at 4°C. After final washing, samples were transferred into Vectashield Mounting Medium (Vector Laboratories, Inc.) and cleared overnight at 4°C. For mounting, small pieces of plasticine were fixed to the corners of cover slips, acting as spacers that prevent squeezing of objects.

### Applied antibodies and antisera

*Primary antibodies*: A monoclonal antibody against acetylated alpha-tubulin (mouse mab 6–11 B-1, Sigma, #T6793, dilution 1∶100) was used to visualize major parts of the cytoskeleton and thereby assess cell shapes and cell extensions. As alternative to acetylated alpha-tubulin labeling, a monoclonal antibody against tyrosinated alpha-tubulin (mouse mab TUB-1A2, Sigma, #T9028, dilution 1∶500) was applied. In order to label cells undergoing mitosis, an IgG fraction of a rabbit antiserum against phosphorylated histone H3 [pSer^10^] (Sigma, #H0412, dilution 1∶200) was used. PH3 labeling was always performed in combination with a nuclear counterstain, and thus co-localization of PH3 and DNA could be confirmed and reliable identification of advanced mitosis stages (telophase) with weak PH3 labeling was made possible. For further characterization of the cellular sub-structures targeted by the applied primary antibodies see Brenneis et al. [53].

*Secondary antibodies*: Primary antibodies were targeted with fluorochrome-coupled secondary antibodies (*α* mouse IgG (H+L) affini pure Cy3, goat mab, Jackson Immunoresearch/Dianova, #115-165-003, dilution 1∶200/*α* rabbit IgG (H+L) Alexa Fluor 488, goat mab, Invitrogen Molecular Probes, #A11038, dilution 1∶400–1∶500).

### Confocal laser-scanning microscopy and data analysis

Image stacks were taken with a Leica DM IRE2 confocal laser-scanning microscope equipped with a Leica TCS SP2 AOBS laser-scan unit. Depending on the intended z-resolution, step sizes from 0.5 µm to 2.5 µm were chosen between successive scanning planes. Based on the emission characteristics of the applied fluorochromes, a combination of UV laser (405 nm wavelength → Hoechst), argon laser (488 nm wavelength → Alexa Fluor 488) and helium-neon laser (543 nm → Cy3, TRITC, FM 1-43FX) was selected for the recordings.

Analyses of the data were performed with the 3D reconstruction program ‘Imaris’ (Bitplane AG, Switzerland, Version 7.0.0). Within the ‘Surpass mode’ of this program, 3D volumes are generated from the recorded image stacks. A volume can be rotated in every spatial dimension and zoomed in and out. It is shown either in the default ‘Maximum intensity projection’ (MIP) or alternatively in the ‘Blend’ option, which renders scanned structures non-transparent and thus facilitates evaluation of the external shape of an object. Additional tools were used for further analysis:

(1)To virtually remove ‘non-target’ regions that obstruct the view of more interiorly located sub-structures of interest ‘Clipping planes’ were applied.

(2)Counts of the overall cell numbers within hemi-neuromeres were performed with the ‘Spots’ tool in combination with an ‘Ortho-slicer’. Both tools together allow systematic manual marking of structures (here nuclei) coupled to an automatic ‘blind’ counting of applied spots.

(3)Nucleus measurements in the (nascent) apical cell clusters of walking leg segment 1 were performed with the ‘Measurement Points’ tool. Measurements were conducted independently in at least two specimens of the same developmental stage. Ten nuclei were measured in each hemi-segment, i.e., twenty nuclei per specimen. Nuclei were measured along their elongated axis.

(4)The ‘Extended section mode’ was used for more detailed analysis of the spatial extensions and relationships of sub-structures. It allows simultaneous visualization of virtual transversal, horizontal and sagittal sections with individually definable thickness (via inclusion of a variable number of images).

### Histology

Bouin-fixed specimens were embedded in the plastic resin Technovit 7100 (Kulzer Histo-Technik) according to the manufacturer's standard protocols. Semi-thin sections (1.5 µm) were cut with a Microm HM 355 microtome, stretched at 60°C on a heating plate and stained in a first step with methylene blue-azure II solution, followed by a counterstain in basic fuchsin solution. Sections were embedded in Roti-Histokitt (Roth) under cover slips. Photographs of selected sections were taken with a Zeiss Axioskop 2 plus microscope equipped with a digital camera (Zeiss AxioCam HRc).

### Scanning electron microscopy

PFA/SW-fixed and PBS-stored specimens were dehydrated in a graded ethanol series (15%, 30%, 50%, 60%, 70%, 80%, 90%, 96%, 2×100%, each step at least 1 h) critical point-dried (using a Bal-Tec CPD 030) and sputtered with gold (using a Bal-Tec SCD 005). Micrographs were taken with a Zeiss LEO 1430.

### Data presentation

Global contrast and brightness values of some of the images were adjusted using Adobe Photoshop CS3. Figures were compiled in Adobe Illustrator CS3. If not stated otherwise, anterior is (1) to the top in all ventral or dorsal aspects and in horizontal sections and (2) to the left in lateral aspects and sagittal sections. In anterior or posterior aspects and transverse sections, dorsal is to the top.

Short movies (avi-format) were generated in Imaris, using the ‘Animation’ mode. They were down-sized to compressed avi-files with the freeware FormatFactory (Version 2.96).

## Results

### Antero-posterior gradient during post-embryonic VNC development

**Figure 1:**
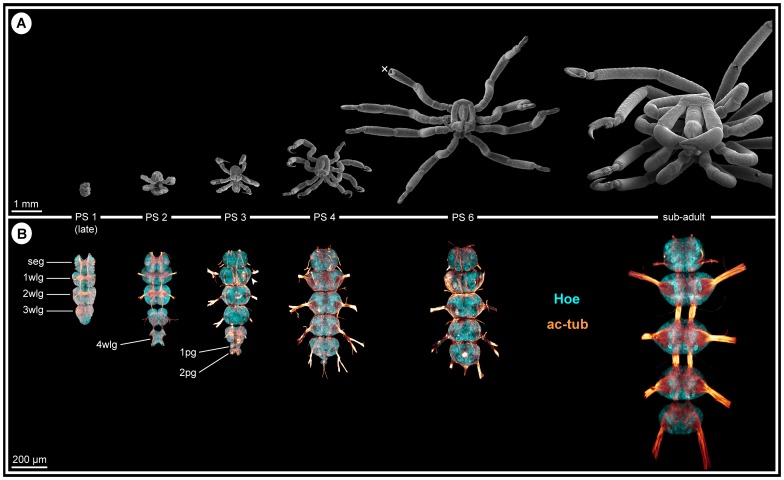
Overview of post-embryonic stages of *Pseudopallene* sp. and corresponding anatomy of the VNC (PS 1 – sub-adult). **A**: SEM micrographs (up to scale), ventral view. Overall body size increases considerably during post-embryonic development. White cross marks damaged distal part of walking leg 1 in PS 6. **B**: Anti-acetylated tubulin labeling (orange) with Hoechst counterstain (cyan). Imaris Surpass mode (volume, MIP). Images up to scale. New segmental ganglion anlagen are added during the first stages of post-embryonic development. The overall size of the VNC increases notably, although not nearly as dramatically as for overall body size. Abbreviations: pg  =  posterior ganglion anlage, PS  =  post-embryonic stage, seg  =  sub-esophageal ganglion, wlg  =  walking leg ganglion (anlage).

As is characteristic for all representatives of the Callipallenidae, *Pseudopallene* sp. has an extended embryonic development. Accordingly, it does not hatch as a minute protonymphon larva with a proboscis and just three larval limb pairs as many non-callipallenid pycnogonids (e.g. [90]–[92]) but instead as a further advanced stage that bears already the elongate limb buds of two walking leg pairs [80], [81]. During embryonic morphogenesis of *Pseudopallene* sp., ten morphologically defined stages have been distinguished [80]. The subsequent anamorphic development encompasses six post-embryonic stages (PSs), which are separated by intermittent molts, an immature sub-adult period (very likely including several molts) and finally the mature adult [81] (see Fig. 1A for some of the stages).

In line with the anamorphic character of post-embryonic development, the VNC of the hatching post-embryonic stage (PS 1) is still incomplete and new segmental ganglion anlagen are added with ongoing development (Fig. 1B). As a consequence, a marked developmental gradient is encountered between the anlagen of sub-esophageal ganglion ( =  palpal and ovigeral neuromeres) and walking leg ganglia 1 and 2, which have been already formed during embryogenesis [53], and those of walking leg ganglia 3 and 4, which develop mainly during post-embryonic development (Fig. 1B). Neurogenesis in these ‘post-embryonic’ ganglion anlagen starts prior to the protrusion of the corresponding limb buds. They represent morphologically distinct units already before external demarcation of the corresponding segment borders has begun. Accordingly, initiation of neurogenesis of walking leg neuromere 3 takes already place during late embryogenesis, whereas the primordium of walking leg 3 is only developed in PS 1 [81]. In PS 2, a well-defined ganglion anlage of walking leg 3 is present (Fig. 1B), but the external segment border between walking leg segments 2 and 3 is still lacking and is present only in the following PS 3 [81]. A similar sequence of developmental events is found in walking leg segment 4.

### Two additional posterior ganglion anlagen

**Figure 2:**
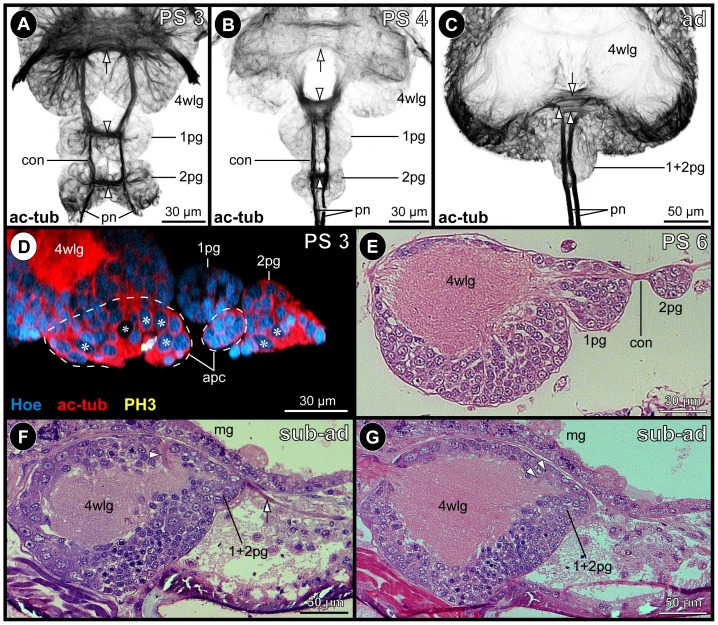
Development and fusion of the posterior ganglion anlagen of *Pseudopallene* sp. Acetylated tubulin (black, red)- and PH3 (yellow)-labeled VNCs with Hoechst (blue) counterstain (**A–D**) and histological sections (**E–G**). **A–C**: Ventral views of the posterior ganglion anlagen, Imaris volume (MIP). Clipping planes have been applied to remove non-target structures in more dorsal and ventral position, only acetylated tubulin labeling shown. Arrowheads mark commissures of posterior ganglion anlagen. Arrow marks postero-ventral commissure of walking leg ganglion 4. Note size decrease of the posterior ganglion anlagen during the fusion process. The paired connectives and proctodeal nerves approach their contra-lateral counterpart medially. **D**: Optical sagittal section through walking leg ganglion 4 and posterior ganglion anlagen in PS 3. Dashed outlines highlight (nascent) apical clusters of walking leg ganglion 4 and posterior ganglion anlage 1, the latter being already detached from the apical ectoderm. Note spindle-shaped cells at the apical side of posterior ganglion anlage 2. Asterisks mark large NSCs that are not in division. **E**: Sagittal section through walking leg ganglion 4 and posterior ganglion anlagen in PS 6. All ganglion anlagen are completely detached from the apical ectoderm. The posterior ganglion anlage 1 has started to fuse with walking leg ganglion 4. **F&G**: Sagittal sections through walking leg ganglion 4 in a sub-adult. The posterior ganglion anlagen and walking leg ganglion 4 are completely fused. A dorsal neural sheath (arrowhead in **F**) indicates the fusion line between them. The two commissural tracts (arrowheads in **G**) of the posterior ganglion anlagen lie closely spaced posterior to the dorsal sheath. White arrow (**F**) points at the posteriorly extending proctodeal nerve. Abbreviations: ad  =  adult, apc  =  apical cell cluster, con  =  connective, mg  =  midgut, pg  =  posterior ganglion anlage, pn  =  proctodeal nerve, PS  =  post-embryonic stage, sub-ad  =  sub-adult, wlg  =  walking leg ganglion.

Between early PS 2 and PS 3, two small posterior ganglion anlagen develop posterior to walking leg ganglion 4 (Figs. 1B; 2A,D). In PS 3, they are each characterized by a compact commissure similar to the early walking leg ganglion anlagen [53] and are inter-connected by longitudinally spanning connectives (Fig. 2A). A paired longitudinal neurite bundle, the proctodeal nerve, extends posteriorly from the second posterior ganglion anlage (Fig. 2A–C) and enters the anal tubercle. Neither segmental, nor inter-segmental nerves are developed. Already in PS 4, both ganglion anlagen have decreased in size (Fig. 2B). They have approached walking leg ganglion 4 more closely, the connectives between the latter and the first posterior ganglion anlage being more compact and significantly shortened (Fig. 2B). As a consequence, the commissure and small neuropil of the first posterior ganglion anlage has moved closer to the neuropil of walking leg ganglion 4. Furthermore, the paired connectives spanning between both posterior ganglion anlagen as well as the posteriorly emanating proctodeal nerves have approached medially (Fig. 2B). This anterior shift of the posterior ganglion anlagen continues and a partial fusion with walking leg ganglion 4 sets in. In PS 6, both posterior ganglion anlagen are still recognizable as separate units (Fig. 2E), whereas they are eventually fused with the postero-dorsal side of walking leg ganglion 4 in sub-adults and adults (Fig. 2C,F,G). Dorsal to the neuropil of the resulting composite ganglion, a prominent neural sheath still separates the somata of the two ontogenetically distinct structures (Fig. 2F). By contrast, their delimitation has become impossible ventral to the neuropil. Two closely approached, short commissures traverse the midline far dorsally and directly posterior to the prominent neural sheath (Fig. 2C,G). Presumably, they represent the commissures of the posterior ganglion anlagen, which have been displaced anteriorly during the fusion process. This is supported by the complete lack of any further posterior commissure, the paired proctodeal nerve being the only further caudally extending neurite bundle (Fig. 2C,F).

### Cell counts during post-embryonic development

**Figure 3:**
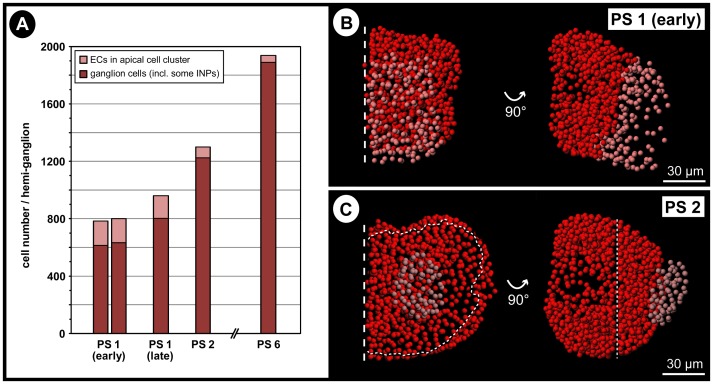
Cell counts in the developing walking leg ganglion 1 of *Pseudopallene* sp. (PS 1 – PS 6). Each bar stands for a single analyzed specimen. Ganglion cells are shown in dark red. Cells of the (nascent) apical cell cluster are labeled in light red. **A**: Overview of counted cell numbers per hemi-ganglion in different stages. Note considerable increase of ganglion cell number in the course of development with a simultaneous decrease of cell numbers in the apical cell cluster. **B&C**: Spots-model of selected hemi-ganglion counts of PS 1 (early) and PS 2. Ventral view on the left side, medial view on the right side. Note the compaction of the (nascent) apical cell cluster (light red) after its complete detachment from the ventral ectoderm in PS 2. Abbreviations: EC  =  ectodermal cell, INP  =  intermediate neural precursor, PS  =  post-embryonic stage.

Apart from the two posterior ganglion anlagen, all ventral ganglia – including those that were differentiated during embryogenesis – exhibit a constant increase in overall volume throughout post-embryonic development. In theory, different processes can lead to an increase of ganglion volume, including *(i)* differentiation and growth of the ganglionic neuropil, *(ii)* size increase of all or some neuronal somata, *(iii)* addition of new cell material into the ganglia, or *(iv)* a combination of these three factors. To test whether neurogenesis is persisting into the post-embryonic phase, nuclei counts were performed. In continuation of cell counts carried out on embryonic stages [53], nuclei in hemi-ganglia of walking leg segment 1 were counted in early and late PS1 as well as in PS 2 and PS 6 (Fig. 3; Movie S1). The results demonstrate a considerable increase in cell numbers per hemi-ganglion during post-embryonic development, their number more than tripling from less than 600 in the last embryonic stage 10 [53] to approximately 1900 in PS 6 (Fig. 3A). Accordingly, ongoing neurogenesis is confirmed as one factor contributing to post-embryonic ganglion growth.

### Neurogenesis in the VNC of PS 1

#### Formation of ventral ectodermal pits

**Figure 4:**
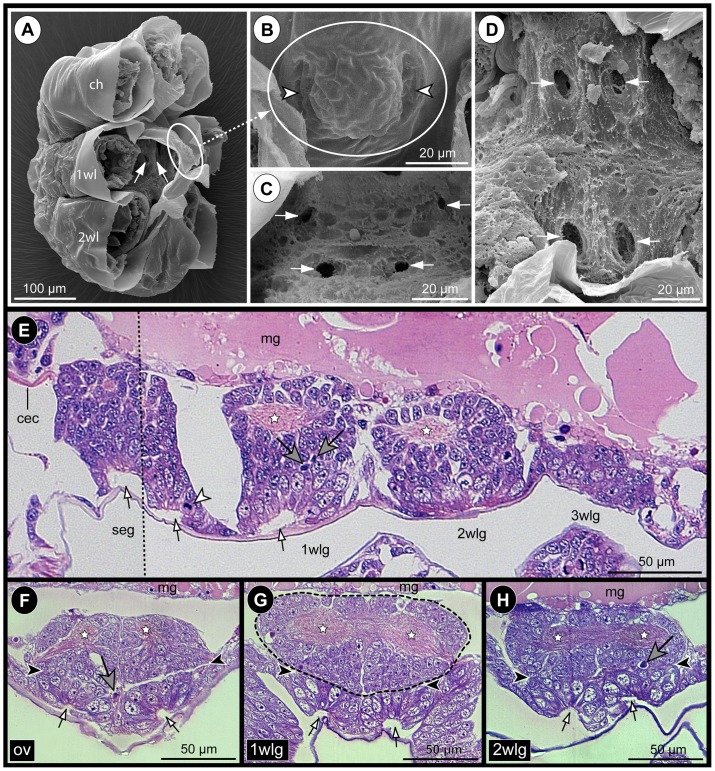
Apical pits and general VNC structure in PS 1 of *Pseudopallene* sp. SEM micrographs (**A–D**) and histological sections (**E–H**). Stars mark ganglionic neuropil. White arrows indicate lumen of apical pits. Big gray arrows mark pycnotic bodies at the basal side of the nascent apical cell clusters. **A**: Lateral view of PS 1, distal portions of proboscis, chelifores and walking leg anlagen removed, anterior to the top, dorsal to the left. Arrows point to the apical ventral pits in walking leg segment 1 under detached cuticle. **B**: Detail of cuticle of walking leg segment 1, ventral view. Cuticular depressions (arrowheads) correspond in position to the underlying ectodermal pits. **C&D**: Ventral detail of apical pits in the palpal and ovigeral segments (**C**) and in walking leg segments 1 and 2 (**D**). The epidermis covers already the complete ventral side except for the segmentally paired pits (arrows). **E**: Composite sagittal section of the VNC of early PS 1 (stippled line indicates border of two images). Sections lie lateral to the midline region. The palpal and ovigeral neuromeres are already fused to form the sub-esophageal ganglion. The apical pit in walking leg segment 2 is not in the plane of the section. The less developed ganglion anlage of walking leg segment 3 still lacks a distinct apical pit. White arrowhead highlights a NSC division. **F–H**: Transverse sections through the ventral ‘embryonic’ ganglion anlagen of a PS 1 specimen slightly older than the one shown in **E**. Note distinct separation (black arrowheads) of the nascent apical cell clusters and the underlying hemi-ganglion anlagen proper (stippled outline in **G**). Abbreviations: cec  =  circum-esophageal connective, ch  =  chelifore, mg =  midgut, GC  =  ganglion cell, ov  =  ovigeral neuromere, seg  =  sub-esophageal ganglion (anlage), wl  =  walking leg (segment), wlg  =  walking leg ganglion (anlage).

**Figure 5:**
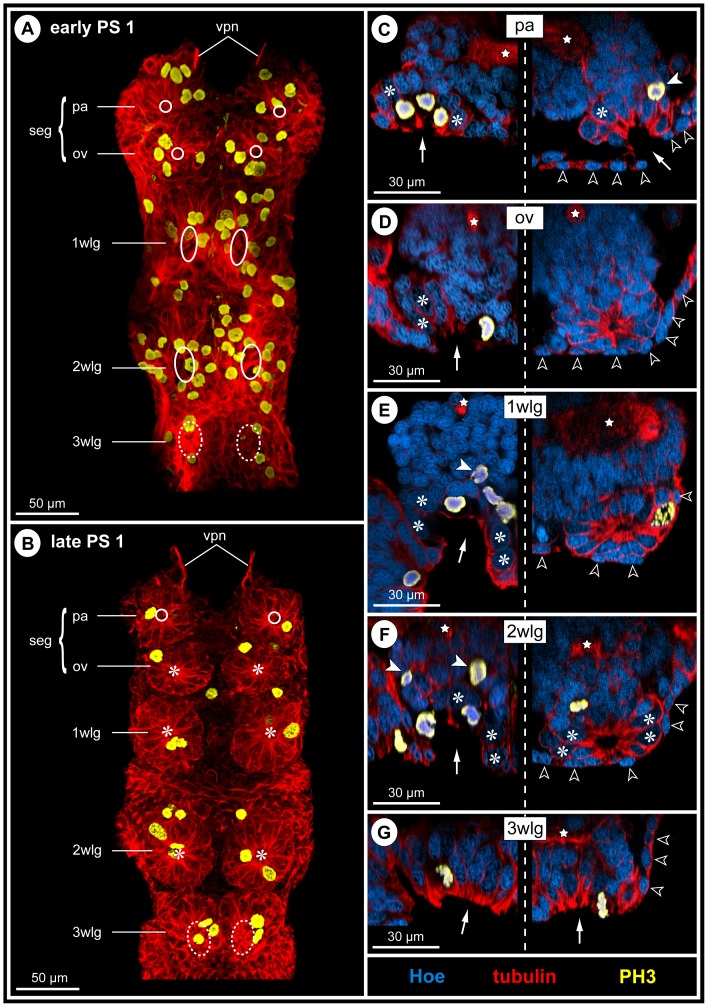
Apical cluster detachment and mitotic activity during PS 1 of *Pseudopallene* sp. **A&B**: Ventral overview of the VNC in early and late PS 1. Imaris volumes (MIP) of acetylated tubulin (**A**) and tyrosine-tubulin (**B**) immunolabeling (both red) and with additional PH3-staining (yellow). White circles and ovals mark apical segmentally paired pits. Stippled ovals indicate shallow apical invagination of the ganglion anlage in walking leg segment 3. Asterisks indicate central cavity of completely detached apical cell clusters. Note decrease of overall mitotic activity from early to late PS 1. **C–G** Apical pits and detached apical clusters in the VNC. Optical transverse sections of tubulin (red)- and PH3 (yellow)-labeled embryos with Hoechst (blue) counterstain. Composite images in which the left side shows early PS 1 (acetylated tubulin) and the right one the corresponding region in late PS 1 (tyrosinated tubulin). Arrows point at the lumen of the apical pits or shallow invaginations. Open white arrowheads indicate small flattened epidermis cells. Asterisks label selected large NSCs (not in division). Filled white arrowheads mark sub-apical divisions of INPs. Stars label neuropil or axonal pathways. Note decrease of mitotic activity from early to late PS 1 and the fully detached apical clusters with central cavity in three segments (ov-2 wl) in late PS 1. Abbreviations: ov  =  ovigeral neuromere, pa  =  palpal neuromere, PS  =  post-embryonic stage, seg  =  sub-esophageal ganglion, vpn  =  ventral proboscis nerve, wlg  =  walking leg ganglion (anlage).

In early PS 1, the ventrally protruding chelifores and the unarticulated limb buds of walking leg 1 and 2 impede an unhindered view at the ventral ectoderm. After manual dissection of these limbs, the cuticular cover of the ventral ectoderm is revealed, being only loosely attached to the underlying tissue (at least in scanning electron microscopical preparations, Fig. 4A). Underneath the cuticle, paired apical pits with a clearly defined, sharp apical rim are present in the ventral ectoderm of the palpal and ovigeral segments and walking leg segments 1 and 2 (Figs. 4A,C–H; 5D–F; Movie S2). These conspicuous pits are the result of a continuing deepening of apical invaginations that have been formed in the ‘embryonic’ ganglion anlagen during late embryonic stages [53]. The cuticle regions covering the ventral pits frequently exhibit a corresponding inwards directed depression (Fig. 4B). Due to the antero-posterior developmental gradient, the walking leg ganglion anlage 3 of early PS 1 shows only a pair of slight apical depressions (Fig. 5G).

#### Structure of the VNC of PS 1

**Figure 6:**
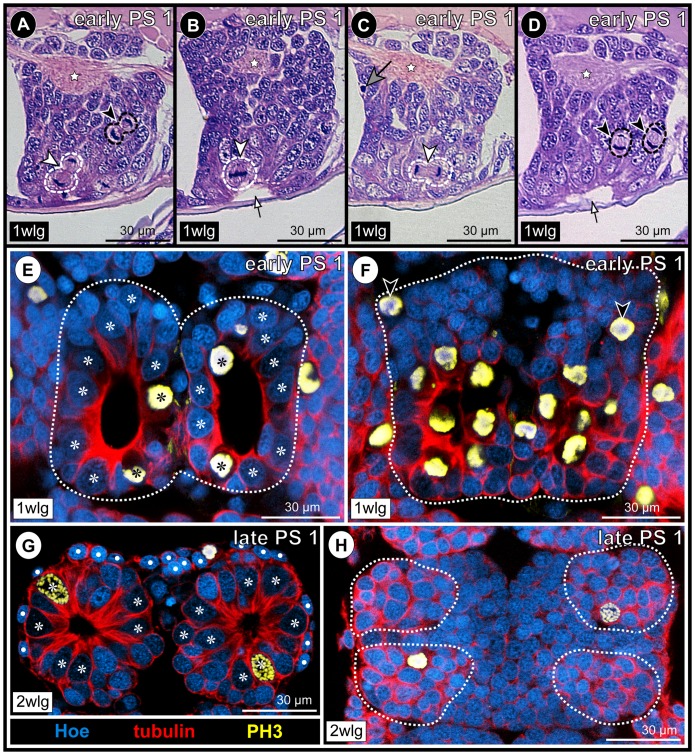
Mitotic activity of apical NSCs and sub-apical INPs in walking leg ganglia 1 and 2 during PS 1 of *Pseudopallene* sp. Histological sections (**A–D**) and optical sections of tubulin (red)- and PH3 (yellow)-labeled specimens with Hoechst (blue) counterstain (**E–H**). **A–D**: Sagittal sections at different levels through walking leg ganglion 1 in early PS 1. Stars mark ganglionic neuropil. Arrows indicate lumen of apical pits. White arrowheads highlight divisions of selected NSCs, the morphological asymmetry of the divisions being visible in late mitotic stages (**A**,**C**). Black arrowheads mark symmetrical divisions of sub-apical INPs (**A**,**D**). Big gray arrow marks pycnotic body (**C**). **E–H**: Horizontal sections through apical pits in early PS 1 and detached apical cell clusters and streams in late PS 1. Acetylated tubulin (**E,F**) and tyrosine tubulin (**G,H**) labeling. Stippled outlines mark extensions of nascent apical clusters and ganglion anlagen proper (**E**,**F**) or anterior and posterior cell streams (**H**). Asterisks label NSCs. **E**: Walking leg ganglion 1, section slightly basal to the epidermal cells covering the rim of the pit. **F**: Walking leg ganglion 1, section through the bottom of the pit being characterized by massive proliferation of the lining NSCs (bottom half of image). Anteriorly, open arrowheads indicate divisions of INPs. At this stage, each nascent apical cluster extends across the postero-ventral side of the corresponding hemi-ganglion proper. **G**: Walking leg ganglion 2, section through detached apical cell clusters at the level of the central cavities. Note size differences of NSCs and the smaller intermingled cells with more brightly stained nuclei. White spots label epidermis cells. **H**: Walking leg ganglion 2, section through the more defined anterior and posterior cell streams that extend into the ganglion proper. Note INP divisions within the streams. Abbreviations: PS  =  post-embryonic stage, wlg  =  walking leg ganglion (anlage).

In early PS 1, the ganglion anlagen of the VNC represent compact cell agglomerations that are positioned directly ventral to the still yolk-filled midgut (Fig. 4E). Up to walking leg ganglion 2, the developing neuropil core of each hemi-ganglion anlage is easily distinguishable in its dorsal half (Figs. 4E–H; 6A–D). The somata of the neurons contributing to the neuropil form a surrounding cortex. The minimal thickness of the cortex is found at the dorsal side, where the neuropil is only partially covered by one or maximally two somata layers. Large neural stem cells (NSCs) are present at the apical (i.e., ventral) side of each hemi-ganglion anlage, lining the interior of the paired pits (Figs. 4E–H; 5C–F; 6E,F; Movie S2; [53]). The sharp apical rim of each pit is formed by distinctly smaller epidermis cells.

#### Separation of ganglion anlagen proper and nascent apical cell clusters

With ongoing development towards late PS 1, the apical portion of each ‘embryonic’ hemi-ganglion anlage starts to separate from the more basal portion, thus forming a nascent apical cell cluster (that still houses the large NSCs) and the underlying hemi-ganglion anlage proper containing the differentiating neurons and glial cells. A distinct separation line between nascent apical cluster and underlying hemi-ganglion anlage proper becomes recognizable (Fig. 4F–H; Movie S2), both sub-structures being sometimes even slightly set off from each other (Fig. 4F). Along the forming separation line, scattered pycnotic bodies are observable (Fig. 4E,F,H), which might point to an involvement of apoptosis in the separation process. However, anteriorly and posteriorly each apical cluster stays confluent with the underlying hemi-ganglion anlage proper. Apically, the lumen of the deep pits in the nascent apical clusters starts to diminish.

#### Cell proliferation in nascent apical cell clusters and connecting cell streams

**Figure 7:**
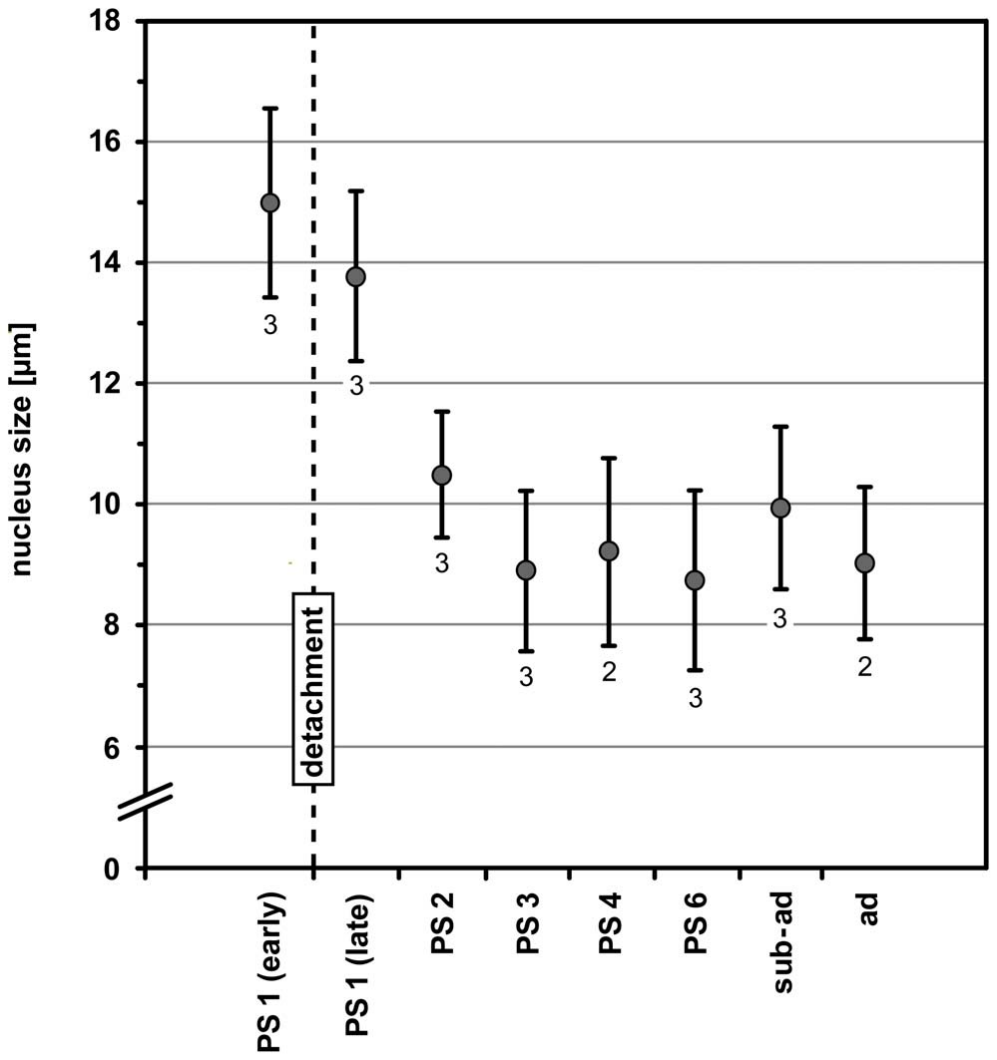
Nucleus measurements in the apical cell clusters of walking leg ganglion 1 of *Pseudopallene* sp. (early PS 1 – ad). The largest nuclei encountered in the (nascent) apical cell clusters have been measured along their elongated axis. Arithmetic means are shown, bars represent standard deviation. Small numbers below the values give the number of specimens analyzed per developmental stage. In early PS 1 the apical pit is still open and lined by numerous large NSCs with nuclei measuring about 15 µm. After apical closure of the pit and detachment of the apical cell clusters (stippled vertical line), nucleus size (and cell size) in the clusters drops considerably to mean values lower than 10 µm. Already from PS 2 onwards NSCs are not reliably identifiable any longer based on morphological characteristics alone. Abbreviations: ad  =  adult, PS  =  post-embryonic stage, sub-ad  =  sub-adult.

In early PS 1, the nuclei of the NSCs lining the pits measure about 15 micrometers along their elongated axis (Fig. 7), which is comparable to late embryonic stages [53]. Considerable mitotic activity is found in the nascent apical cell clusters and the apical part of the hemi-ganglion anlagen proper (Figs. 5A,C–F; 6A–F; Movie S2). To a large part, cell divisions are attributable to the divisions of the apical NSCs (Figs. 5C–E; 6A–C,E,F) but also to sub-apical divisions of smaller intermediate neural precursors (INPs, Figs. 5E,F; 6A,D,F; Movie S2). Advanced cell division stages of the apical NSCs show morphological asymmetry, whereas divisions of the sub-apical INPs appear to be morphologically symmetrical (Fig. 6A,C,D; [53]). INP divisions are mostly encountered in the anterior and posterior connecting regions where the nascent apical cluster and the hemi-ganglion anlage proper are still confluent. The restriction of cell divisions to apical and sub-apical layers coupled to the simultaneous increase of cell numbers in the underlying hemi-ganglion anlage proper (which is devoid of cell proliferation) suggest the anterior and posterior connecting regions to be pathways (‘cell streams’) along which apically produced cell material moves basally.

#### Detachment of apical cell clusters and formation of defined cell streams in late PS 1

In late PS 1, the ventral ectoderm of walking leg segment 3 exhibits still a pair of shallow apical depressions (Fig. 5G). By contrast, the ventral ectoderm of the palpal segment up to walking leg segment 2 shows either paired tiny ‘holes’ (e.g. palpal segment, Fig. 5B,C) or no signs of the previous deep pits any longer (ovigeral segment – walking leg segment 2, Fig. 5B,D–F). This is due to a proceeding detachment of the nascent apical cell clusters from the epidermal cell layer. The cells that have been lining the pit of each nascent apical cluster in early PS 1 approach each other centrally during the detachment process, leading at first to a narrowing of the apical opening and eventually to its complete closure. As a consequence of these cell movements, a small cavity is formed in the center of each cluster. The original apical poles of the cells are oriented internally, i.e., towards the central cavity (Figs. 5D–F; 6G; Movie S3). The small epidermal cells that have been apically encircling each pit's rim follow the centrally directed movement but are not incorporated into the internally detaching cluster. Instead, they remain in apical position and form a continuous thin epidermal layer as soon as the apical opening has been closed (Fig. 5C–F; Movie S3). Accordingly, the detachment of the apical cell clusters results in the final and complete spatial separation of the ventral ectodermal cells with a neural fate and those with epidermal fate. Notably, this final step of the internalization of the ganglion anlagen does not seem to be characterized by a ‘proper’ active overgrowing by the future ventral epidermis. Rather, it appears to be more passive, being driven by the steady approach of the apical poles of the cells in the nascent apical clusters of the hemi-ganglion anlagen.

Compared to early PS 1, the anterior and posterior confluent cell regions connecting the apical cluster and corresponding hemi-ganglion anlage proper gradually begin to represent more defined streams (Fig. 6H). Each stream is characterized by intense tubulin labeling and is still several cells wide.

#### Decreasing cell size and reduced mitotic activity in apical cluster-stream-systems

In late PS 1, the apical cell clusters still comprise larger NSCs with weakly Hoechst-labeled nuclei, interspersed with smaller cells with more brightly stained nuclei (e.g. Figs. 5E,F; 6G; Movie S3). The majority of cells are of spindle- to flask-shaped appearance, extending cell processes towards the central cavity of a cluster. Notably, nucleus measurements in the apical clusters of walking leg ganglion 1 indicate a slight decrease in the size of the apical NSCs compared to the preceding stage (Fig. 7). This decrease is accompanied by reduced mitotic activity in late PS 1 (Fig. 5B). Mitoses are found in the apical cluster-stream-systems (CSSs), relating either to the NSCs within the clusters, or alternatively to smaller, more sub-apical cells that lie often in the clusters’ periphery, close to the origin of the anterior and posterior cell streams or within the cell streams themselves (Figs. 5E,F; 6G,H).

### Neurogenesis in the VNC of PS 2

#### Structure of the VNC of PS 2

**Figure 8:**
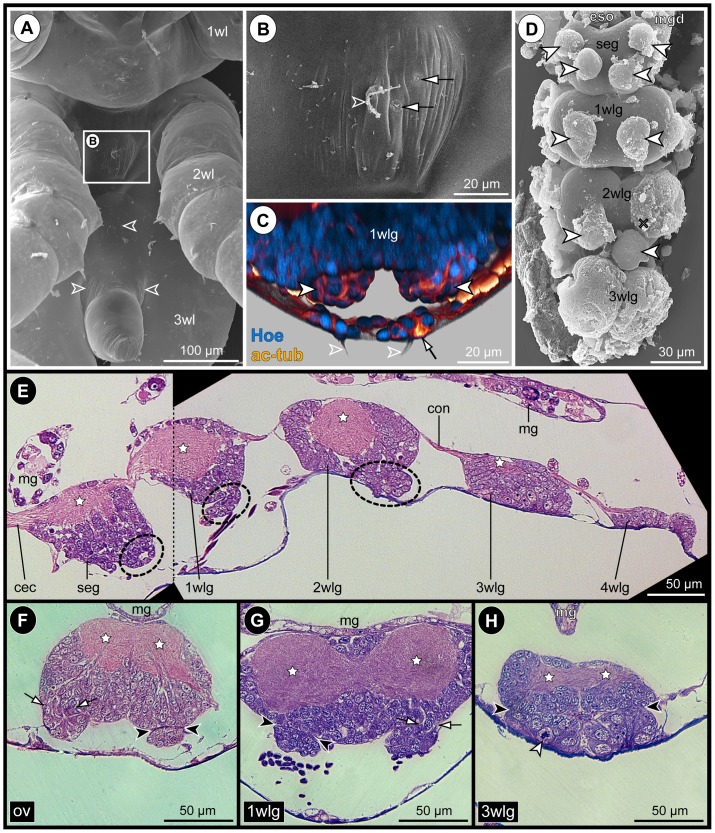
Sensory setae, epidermal glands and VNC structure in PS 2 of *Pseudopallene* sp. SEM micrographs (**A**,**B**,**D**), acetylated tubulin (orange) labeling with Hoechst (blue) counterstain (**C**) and histological sections (**E–H**). **A**: Walking leg segment 1 to anal tubercle, ventral view. Small fields of cuticular folds (arrowheads) indicate location of underlying segmental ganglion anlagen. **B**: Detail of area highlighted in **A**. The cuticle bears bifurcate setae (arrowhead) and minute slit-openings of epidermal glands (arrows). **C**: Anterior *in-situ* view of walking leg ganglion 1. Imaris volume (blend), clipping planes used to reveal target structures. The apical cell clusters (filled arrowheads) are completely detached from the ventral epidermis with no connection to cells of the slit glands (arrow) or setae (open arrowheads). Longitudinal muscles (orange-yellowish bands) are in part located between apical clusters and ventral epidermis. **D** Ventral view of VNC (primordium of 4 wlg missing). Arrowheads indicate detached apical clusters. Cross marks the original position of the left apical cluster of 2 wlg, having been posteriorly displaced during dissection. The nascent apical clusters of 3 wlg still cover its entire ventral side. **E–H**: Stars mark ganglionic neuropil. Arrows indicate regions where cell streams enter underlying ganglion proper. Black arrowheads indicate separation lines between (nascent) apical clusters and underlying ganglia proper. **E**: Composite sagittal section of the VNC (stippled line indicates border of two images), lateral to the midline region. Stippled ovals highlight detached apical clusters. The cluster of the palpal neuromere lies not in the plane of the section. Note apical attachment of the less-developed anlagen of ‘post-embryonic’ 3 wlg and 4 wlg. **F–H**: Transverse sections through different ventral ganglia (anlagen). Note decreased cell size in more anterior compared to more posterior apical clusters (**F**,**G** and **H**, respectively). White arrowhead in **H** marks NSC division. Abbreviations: cec  =  circum-esophageal connective, con  =  connective, eso  =  esophagus, mg  =  midgut, mgd  =  midgut diverticulum, NSC  =  neural stem cell, ov  =  ovigeral neuromere, seg  =  sub-esophageal ganglion, VNC  =  ventral nerve cord, wl  =  walking leg (bud), wlg  =  walking leg ganglion (anlage).

PS 2 shows most clearly the considerable antero-posterior developmental gradient between the anterior ‘embryonic’ ventral ganglia and the more posterior ‘post-embryonic’ ventral ganglion anlagen. Three different developmental states of the ganglion anlagen can be observed within a single specimen of PS 2 (Fig. 8E).

**Figure 9:**
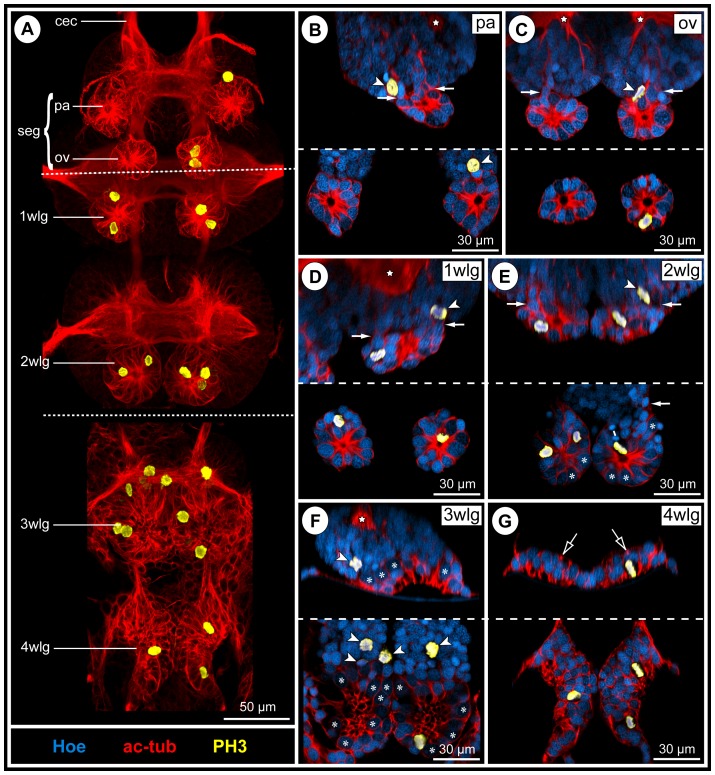
Mitotic activity in detached and nascent apical CSSs in the VNC of *Pseudopallene* sp. (PS 2). Acetylated tubulin (red) and PH3 (yellow) labeling of VNCs with Hoechst (blue) counterstain. **A**: Composite image of VNC, ventral view. Imaris volume (MIP), Hoechst counterstain not shown, stippled lines mark borders of separate images. Note that mitoses in the anterior ‘embryonic’ ganglia (seg-2 wlg) are restricted to the detached apical clusters. **B–F**: Optical sections through detached and nascent apical clusters and underlying segmental ganglia (anlagen). Sagittal (**B**,**D**,**F**) or transverse (**C**,**E**) sections above dashed lines, horizontal sections below dashed lines. Stars indicate ganglionic neuropil. White arrows mark anterior and posterior cell streams. Open arrowheads indicate sub-apical mitoses close to or within the cell streams. Asterisks (**E**,**F**) highlight selected apical NSCs that are not in mitosis. Note tiny central cavities and still detectable differences in cell sizes and nuclear staining intensities in the detached anterior clusters (pa-2 wlg). The paired nascent apical clusters of the anlage of walking leg ganglion 3 are characterized by a nuclei-free apical invagination. **H**: Optical sections through primordium of walking leg ganglion 4. Transverse section above dashed line, horizontal section below dashed line. Arrows indicate basal anlagen of the longitudinal connectives. Note tangential apical mitosis and lack of large NSCs in this early neurogenic phase. Abbreviations: cec  =  circum-esophageal connective, ov  =  ovigeral neuromere, pa  =  palpal neuromere, seg  =  sub-esophageal ganglion, wlg  =  walking leg ganglion (anlage).

(1) The ‘embryonic’ ganglia have gained in overall size and are less compressed than in the preceding stages. Their ganglionic neuropil has increased markedly, occupying a major part of the dorsal half of each ganglion (Fig. 8E–G; Movie S4). The paired apical clusters of all ‘embryonic’ ganglia have completely detached from the epidermis (Fig. 8C–G; Movie S4), representing compact roundish to ellipsoid cell agglomerations at the ventral side of the latter (Fig. 8D). In part, they are distinctly separated from the epidermis by longitudinal muscles (Fig. 8C,E,G; Movie S4).

(2) The anlage of walking leg ganglion 3 is less differentiated. It is already characterized by nascent apical cell clusters that have as yet not separated from the epidermis (Figs. 8D,E,H; 9F), reminiscent of the ‘embryonic’ ventral ganglion anlagen in the preceding PS 1.

(3) The anlage of walking leg ganglion 4 represents only a spatially restricted accumulation of cells, the nuclei of which being arranged in one to two apico-basal levels (Figs. 8E; 9G). Typical for early stages of neurogenesis, the cells are in part flask-shaped, mitoses are found apically and no morphologically distinct NP types can be identified as yet (Fig. 9G; [53]).

In terms of overall size, the antero-posterior gradient of the apical cell clusters is directly opposite to the underlying ganglion anlagen proper, anterior clusters being slightly smaller and more compact than more posterior ones (Fig. 8D).

#### Sensory and glandular structures of the ventral epidermis and their (non-)relation to the apical CSSs

In contrast to the preceding PS 1, the cuticle of the actively moving PS 2 is equipped with numerous bifurcating setae and tiny slit-like pores of epidermal glands. Some of these structures are also covering the ventral side of the trunk, in the regions overlying the ganglion anlagen of the VNC (Fig. 8A–C). Neither in histological sections, nor in analyses of complete stacks of optical sections a connection between the detached apical clusters of the ‘embryonic’ ganglia and the epidermal sensory or glandular structures could be detected (Fig. 8C). Rather, innervation of the sensory setae is accomplished via delicate peripheral nerve branches running within or directly underneath the loose cellular network of the epidermis (data not shown). Hence, no indications for a direct involvement of the apical CCSs in the processing of epidermal sensory input could be found and any role of the apical clusters in the external secretion of the epidermal slit glands can be excluded.

#### Disappearance of large NSCs in the apical cell clusters

The only mitoses in the anterior VNC of PS 2 are encountered in the apical CSSs. (Fig. 9A–E; Movie S4). Mitotic activity seems to have further decreased compared with late PS 1. While some clusters do not show any mitotic profiles, in others one to two mitoses are detectable, but only very rarely more than that were documented (Fig. 9A–E). Although some differences in cell sizes and nuclear staining intensity are still observable within each cluster, these are by far not as significant as in the preceding stages (Fig. 8B–E; Movie S4). Nucleus measurements demonstrate that the cells in the clusters have further decreased in size (10–11 micrometers, Fig. 7). Hence, based on morphological features, NSC identification in the apical cell clusters of the anterior VNC becomes more challenging, whereas they are still easily identifiable in the nascent apical cluster of the more posterior anlage of walking leg ganglion 3 (Figs. 8E,H; 9F). It remains unresolved whether NSCs have completely ceased to exist in the anterior clusters (i.e., have either undergone apoptosis or terminal division) or are only not traceable any longer due to size decrease and the limitations of the applied methods. The observed mitoses prove that at least some of the cluster cells are not (yet) post-mitotic, but unambiguous indications of asymmetrical divisions were not detected. Compared to PS 1, the anterior and posterior streams of the CSSs have further diminished in diameter, being more slender in the more anterior ventral ganglia (Figs. 8F,G; 9B–E; Movie S4).

### Neurogenesis in the VNC of PS 3 to PS 6, the sub-adult and the adult

#### Structure of the VNC from PS 3 to the adult

In PS 3 and PS 4, the ‘post-embryonic’ segmental ganglia gain on the more anterior ones in terms of overall size, cell number and neuronal differentiation, so that in PS 4 all four walking leg ganglia eventually exhibit a corresponding level of development (Fig. 1B). Posterior to walking leg ganglion 4, the two posterior ganglion anlagen are transiently discernible (see above; Figs. 1B; 2).

**Figure 10:**
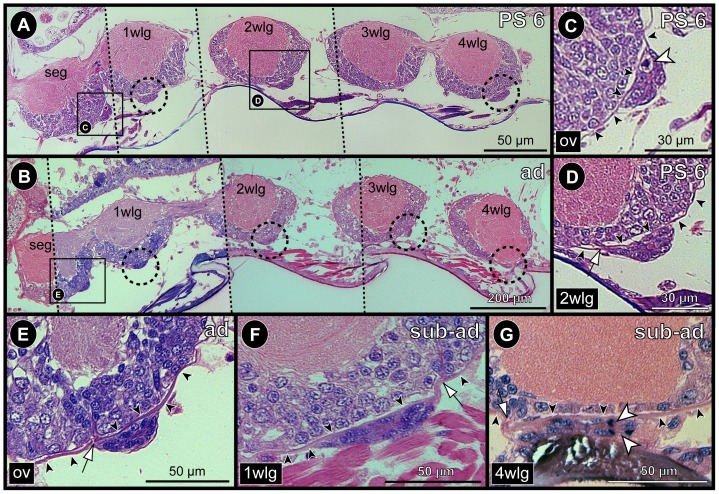
Persistence of apical CSSs into the adult of *Pseudopallene* sp. Sagittal histological sections. Black arrowheads highlight the neural sheath. Arrows indicate fibrous cell streams that penetrate through the neural sheath into the ganglionic somata cortex. White arrowheads mark mitoses in apical cell clusters. **A&B**: Composite overview images of the VNC of PS 6 (**A**) and the adult (**B**). Stippled lines indicate borders between separate images. Adjacent original images show sections at slightly different levels in order to depict more apical cell clusters. Stippled circles highlight apical clusters that are not shown in a separate detail. The palpal cluster is not shown in any overview, owing to its further lateral position (see also Fig. 7D). Note the similar or occasionally darker nuclear staining of the cluster cells compared to differentiated neuronal nuclei. **C&D**: PS 6. **C**: Ovigeral CSS. Note cell division in the apical cluster. **D**: CSS of walking leg ganglion 2. Note the tiny central cavity (stained in pink) in the apical cluster. **E**: Ovigeral CSS in an adult. **F&G**: Sub-adult. **F**: CSS of walking leg ganglion 1. Note elongated shape of the apical cluster. **G**: CSS of walking leg ganglion 4. Note mitotic profiles in the elongated and flattened apical cluster. Abbreviations: ad  =  adult, ov  =  ovigeral neuromere, PS  =  post-embryonic stage, seg  =  sub-esophageal ganglion, sub-ad  =  sub-adult, wlg  =  walking leg ganglion.

From PS 3 to early PS 6, the walking leg ganglia of adjoining segments are touching each other (Fig. 1B), but stay anatomically completely separated by their well developed neural sheaths. In the following late PS 6 (Fig. 10A), sub-adults (Fig. 1B) and adults (Fig. 10B), such a close spatial proximity is only retained between the sub-esophageal ganglion and walking leg ganglion 1. The more posterior ganglia are distinctly separated and inter-connected by paired longitudinal connectives that lack a cortex of neuronal somata but contain elongated, flattened nuclei of glial cells that ensheath separate axon bundles within the connectives (not shown). Up to the adult, the volume of the ganglionic neuropil in the ventral ganglia increases continuously (Figs. 8E; 10A,B). Also the number of GCs is significantly growing during advanced post-embryonic development as shown by our cell counts in the hemi-ganglia of walking leg segment 1 (Fig. 3).

#### Low mitotic activity in persisting apical CSSs

**Figure 11:**
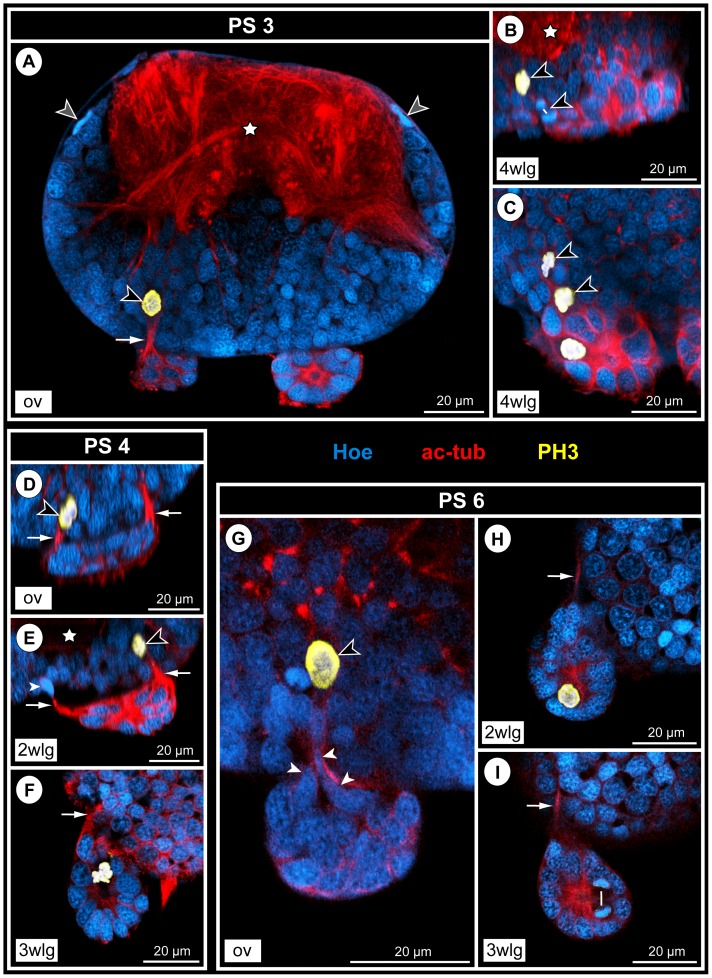
Mitotic activity in detached CSSs during advanced post-embryonic development of *Pseudopallene* sp. Optical sections of acetylated tubulin (red)- and PH3 (yellow)-labeled VNCs with Hoechst (blue) counterstain. White arrows label fibrous anterior and posterior cell streams. Black arrowheads mark sub-apical mitoses close to or within cell streams. White arrowheads indicate oblong nuclei of cells that migrate along the fibrous cell streams. Stars label ganglionic neuropil. Note the absence of distinctly larger cells in the apical clusters. **A–C**: PS 3. **A**: Slightly oblique transverse section through sub-esophageal ganglion at the level of the ovigeral cluster-stream-system (CSS). Note flattened nuclei (gray arrowheads) of glial cells in the neural sheath surrounding the ganglion. **B**&**C**: Sagittal (**B**) and horizontal section (**C**) through walking leg ganglion 4, respectively. Note the still diffuse, not clearly defined cell streams in this posterior walking leg ganglion. **D–F**: PS 4. **D**: Sagittal section through ovigeral CSS. **E**: Sagittal section through CSS of walking leg ganglion 2. **F**: Horizontal section through CSS of walking leg ganglion 3. **G–I**: PS 6. **G**: Transverse section showing detail of ovigeral CSS. Note the narrow neck of the nuclei (arrowheads) of cluster cells that start to extend into the cell stream. **H**: Horizontal section through CSS of walking leg ganglion 4. **I**: Horizontal section through CSS of walking leg ganglion 3. The white line extends between two newly forming nuclei during telophase. The cell division is morphologically symmetrical. Abbreviations: ov  =  ovigeral neuromere, PS  =  post-embryonic stage, wlg  =  walking leg ganglion (anlage).

**Figure 12:**
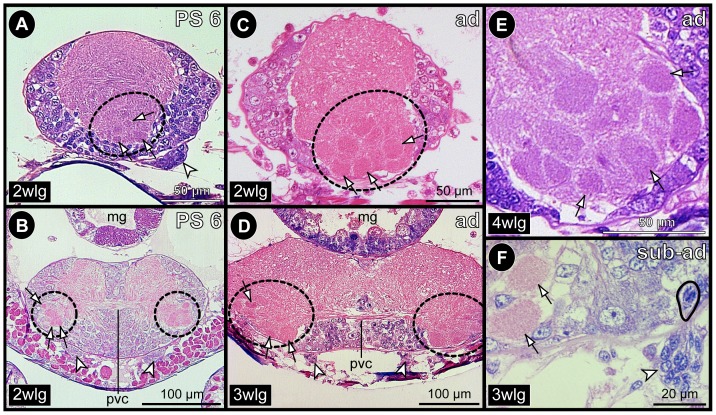
Glomerulus-like neuropils in the VNC of *Pseudopallene* sp. Histological sections through ventral ganglia. Stippled ovals highlight ventro-lateral neuropil regions housing glomerulus-like neuropils (GNs). Arrows point at selected GNs. Arrowheads indicate apical cell clusters. Ganglion identity as indicated in bottom left corner of each image. **A&B**: PS 6. **A**: Sagittal section. **B**: Transverse section. **C–E**: Adult. **C**: Sagittal section. **D**: Transverse section. **E**: Magnification of GN-containing neuropil region, sagittal section. **F**: Sub-adult. Transverse section showing two ventro-lateral GNs and medial to them the apical cell cluster with one of its cell streams, which penetrates the neural sheath. One cell in the slender cell stream is in mitosis (black outline). Abbreviations: ad  =  adult, mg  =  midgut, PS  =  post-embryonic stage, pvc  =  postero-ventral commissure, sub-ad  =  sub-adult, wlg  =  walking leg ganglion.

The apical cell clusters persist as roundish to ellipsoid structures on the ventral side of the ganglia from PS 3 to PS 6 (Figs. 10A,C,D; 11; 12A,B; Movie S5) but often assume a more antero-posteriorly elongated shape in the sub-adult and adult stages (e.g. Fig. 10F,G). They are separated from the ganglia by the neural sheath (Fig. 10C–G). The only connections of an apical cluster into the interior of the respective ganglion are the anterior and posterior streams, which penetrate the neural sheath (Figs. 10D–G; 11; 12F; Movie S5) and have continued to further decrease in diameter in comparison to PS 2. Already from PS 3 on, they represent in all ventral ganglia – except for the slightly less developed walking leg ganglion 4 – just tubulin-positive fibrous strands (Fig. 11; Movie S5). Cluster cells situated close to the cell streams are often characterized by elongated nuclei that are sometimes found to extend into the respective narrow stream (Fig. 11G). This suggests a continuing cell migration into the underlying ganglion. Along the apico-basal extension of the cell streams, only few cells with elongated, often intensely stained nucleus are found (e.g. Fig. 11E; Movie S5). Compared to the early PS 1 and PS 2, even fewer mitoses occur in the VNC, being exclusively restricted to the apical CSSs (Figs. 10C,G; 11; 12F; Movie S5). Frequently, CSSs do not show any mitotic profiles. Judging from the nucleus measurements, cell sizes within the clusters have further decreased, mean nucleus size never exceeding 10 micrometers (Fig. 7).

### Cell numbers in the apical clusters during post-embryonic development

**Figure 13:**
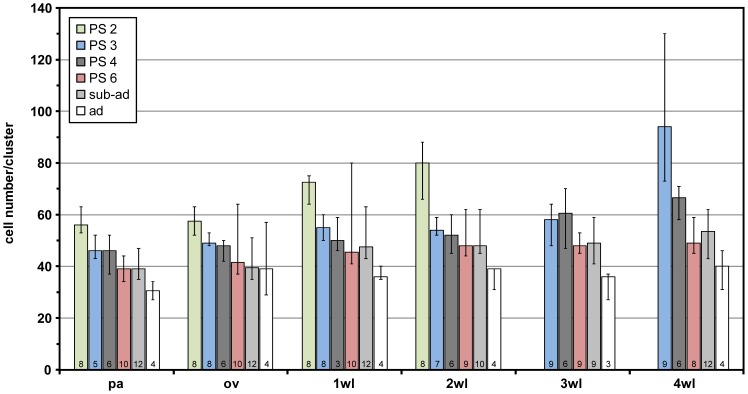
Cell numbers in apical clusters during post-embryonic development of *Pseudopallene* sp. Developmental stages are color-coded as shown in the legend in the upper left corner. Segmental affiliation of the clusters is shown on the x-axis. Each column represents the median of counted cell numbers per cluster and developmental stage. The sample size of counted clusters is shown at the bottom of each column. Sample size differences between different clusters of the same developmental stage relate to cluster damage or loss during dissection. ‘Error’ bars indicate maximum and minimum cell counts. Complete lack of values (PS 2–3 wlg, 4 wlg) is related to the early differentiation state of the respective ganglion anlagen with no clearly separated clusters on the ventral side. Abbreviations: ad  =  adult, ov  =  ovigeral neuromere, pa  =  palpal neuromere, PS  =  post-embryonic stage, sub-ad  =  sub-adult, wl  =  walking leg neuromere.

From PS 2 to the adult stage, the number of cells per apical cluster was determined for each ventral ganglion/ganglion anlage (see Movie S1). Since PS 5 specimens were extremely scarce in the collected material, this stage had to be excluded from the analysis. Due to the compact shape and well defined borders of the apical clusters, cell numbers could be reliably counted in almost all ventral ganglia/ganglion anlagen of the included stages. This excludes only the anlagen of walking leg ganglia 3 and 4 in PS 2, which are not yet showing detached and clearly separated apical clusters. Two main trends can be extracted from the obtained results (Fig. 13).

(1) In specimens of earlier post-embryonic stages (PS 2–PS 4), the clusters of more posterior ganglia/ganglion anlagen generally comprise more cells than the anterior ones. Accordingly, the apical clusters are larger in the more posterior ganglia/ganglion anlagen, which are less developed. This intra-individual antero-posterior trend is more pronounced in the earlier stages (PS 2 and PS 3) and gradually subsides with the diminishing developmental differences between the ‘embryonic’ and ‘post-embryonic’ ventral ganglia.

(2) With ongoing development, a gradual intra-segmental decrease in cell numbers per cluster is detectable. Starting out with more than 50 cells (minimum) per cluster, this number constantly diminishes towards 40 or less in the adult.

This demonstrates that the cell divisions in the apical CSSs do not result in a lasting increase of cell numbers within the clusters themselves. Since no systematic occurrence of pycnotic bodies (as indicators for cell death) was observed within the clusters, it can be concluded that some cells leave the latter during the post-embryonic phase. In light of the simultaneous cell number increase in the completely ensheathed ventral ganglia, these findings lend additional support to the inferred direction of cell movement from apical clusters into the underlying ganglia.

### Glomerulus-like neuropils in the VNC of *Pseudopallene* sp

In histological sections of *Pseudopallene* sp. adults and less distinctly also in PS 6, a number of glomerulus-like neuropils (GNs) were identified ventro-laterally in each walking leg ganglion (Fig. 12). Glomerulus-like neuropils are very characteristic primary centers in the olfactory pathway of arthropods (and other animals). Similar but less voluminous neuropilar structures appear to be present in the sub-esophageal ganglion (data not shown). A detailed description of the exact arrangement, number, shape and architecture of the GNs lies beyond the scope of the present work and will be focus of future investigations. Notably, the apical CCSs and the GNs show close spatial vicinity in the ventral ganglia (Fig. 12A,B,D,F).

## Discussion

### The posterior ganglion anlagen during pycnogonid development - evolutionary implications

During post-embryonic development of *Pseudopallene* sp., two transiently separate ganglion anlagen develop posterior to walking leg ganglion 4. They are most voluminous in PS 3, have already decreased in size in PS 4 and eventually fuse with walking leg ganglion 4 between PS 6 and the sub-adult stage. With our available data, it remains unclear whether cell migration, cell death or a mixture of both phenomena leads to the size decrease of the two posterior ganglion anlagen.

In several previous studies, one or two transiently separate ‘abdominal ganglia’ at the postero-dorsal margin of walking leg ganglion 4 have been described (*Callipallene emaciata* [87], [93]; *C. brevirostris* [82]; *Endeis spinosa* [84], [93]; *Colossendeis proboscidea* [94]; *Nymphon spinosum* [83], [84]; *Achelia laevis* [84]). Dohrn [93] even mentions the separate persistence of these two ganglia in adults of *E. spinosa*, but this claim found no confirmation by Dogiel [84], who reported their complete fusion with the last walking leg ganglion. Hence, it appears likely that Dohrn's description is based on an immature specimen, which he misidentified as adult. In confirmation of our findings on *Pseudopallene* sp. (Fig. 2D), the formation of apical invaginations as well as small apical cell clusters have been reported during development of the posterior ganglion anlagen in *N. spinosum* [83], [84] and *C. emaciata* [87]. Furthermore, Hoek [94] was able to delimit two small dorsal neuropil cores at the posterior side of the extensive neuropil of walking leg ganglion 4 in adults of *Nymphon strömi* and *Boreonymphon robustum*, which he assigned to fused posterior ganglion anlagen. These overall correspondences across five pycnogonid taxa indicate transient posterior ganglion anlagen to be a widespread and probably plesiomorphic developmental feature in crown-group pycnogonids.

Against an evolutionary background, the anal tubercle of crown-group pycnogonids is frequently interpreted as vestige of a formerly segmented posterior trunk region. Especially in the framework of the Chelicerata concept, it has been considered as representing a reduced opisthosoma [95]–[98]. This reduction scenario receives some support from the fact that the Hox-gene abdominal-A, which is expressed in the opisthosoma of spiders [99], [100], has been shown to be highly modified in *Nymphon gracile* [101] and from several fossils that have been placed within the pycnogonid lineage, possessing a posterior-most limbless trunk region with external segmentation or at least traces thereof (*Palaeoisopus problematicus*, *Palaeopantopus maucheri* [102]; *Haliestes dasos* [103]; *Flagellopantopus blocki* [104]). The occurrence of posterior ganglion anlagen during development of extant pycnogonids may be seen as additional evidence corroborating this view. Indications for their serial homology to the walking leg ganglia are *(i)* their position in line with the segmental ganglia of the VNC, *(ii)* their inter-connection to the latter via paired longitudinal connectives (prior to fusion), *(iii)* the establishment of a commissure per posterior ganglion anlage and *(iv)* their apparently similar formation process with paired apical invaginations and briefly persisting apical cell clusters (in nymphonids and callipallenids). Based on this, the posterior ganglion anlagen of extant pycnogonids can be interpreted as vestiges of formerly fully developed segmental ganglia, the segments of which having been largely reduced in the pycnogonid stem-lineage. Remarkably, however, inclusion of the pycnogonid fossils in phylogenetic analyses has placed some of these specimens not in the stem-lineage but rather within the crown-group of pycnogonids [103], [105]. If true, this would indicate independent reduction events of posterior trunk segmentation within crown-group pycnogonids. Yet, owing to low support values, these findings have to be considered with care.

We have shown that the onset of segmental neurogenesis during the anamorphic post-embryonic development of *Pseudopallene* sp. predates the emergence of limb buds or any other morphological signs of segment formation. An identical temporal sequence has been described in earlier studies [82], [106]–[108]. Hence, the formation of posterior ganglion anlagen without differentiation of corresponding segments may be related to a specific sequence in the formation of segmental sub-structures. During pycnogonid evolution, only those differentiation processes subsequent to the initiation of neurogenesis appear to have been completely ‘silenced’ in the posterior-most trunk region. A next step towards a better understanding of this developmental phenomenon would be the investigation of segment polarity genes. This could help to assess how many segment primordia are prefigured in the differentiating hind body region during development. A similar approach has already revealed vestigial segment anlagen in the embryonic pleon of the decapod *Cherax destructor* [109] and in the abdomen of the cirripede *Sacculina carcini* [110].

In this context, the nervous system development and adult neuroarchitecture of ten- and twelve-legged pycnogonid species represents another intriguing field of investigation. Such extra-legged species occur in a disjunct distribution in three different extant pycnogonid taxa (Pycnogonidae, Colossendeidae, Nymphonidae) and in the fossil representative *Pentapantopus vogteli*, which has been placed within crown-group pycnogonids [111]. Most of the extant extra-legged pycnogonids show striking morphological similarities to ‘normal’ eight-legged species, which strongly suggests them to be closely related to the latter [112]. This indicates that additional leg-bearing segments have independently evolved within crown-group Pycnogonida. At the nervous system level, it would be interesting to investigate how many ‘super-numerous’ posterior ganglion anlagen are developed in extra-legged representatives. A negative correlation between the number of additional segmental ganglia and the one of the posterior ganglion anlagen could hint on a reactivation of segment differentiation processes in posterior segment primordia of formerly eight-legged forms.

### Apical CSSs as characteristic feature of a third phase of pycnogonid neurogenesis

In a previous study, we have shown that embryonic neurogenesis of *Pseudopallene* sp. is characterized by two phases [53]. The early first phase features immigration of predominantly post-mitotic GCs from the ventral neuroectoderm, taking place in transiently identifiable cell internalization sites arranged in an at least partially stereotyped arrangement. This is similar to early neurogenesis in euchelicerate and myriapod taxa [26], [30]–[37]. In the second phase of *Pseudopallene* sp., paired apical invaginations are formed in each ventral neuromere and large NSCs with high mitotic activity and an asymmetrical division mode differentiate within these invaginations. Furthermore, an additional type of INP is found directly basal to the apical NSCs. These invaginating ectodermal cell regions are the centers of advanced embryonic neurogenesis and have been somewhat unfortunately called ‘ventral organs’ in previous studies [82]–[87].

**Figure 14:**
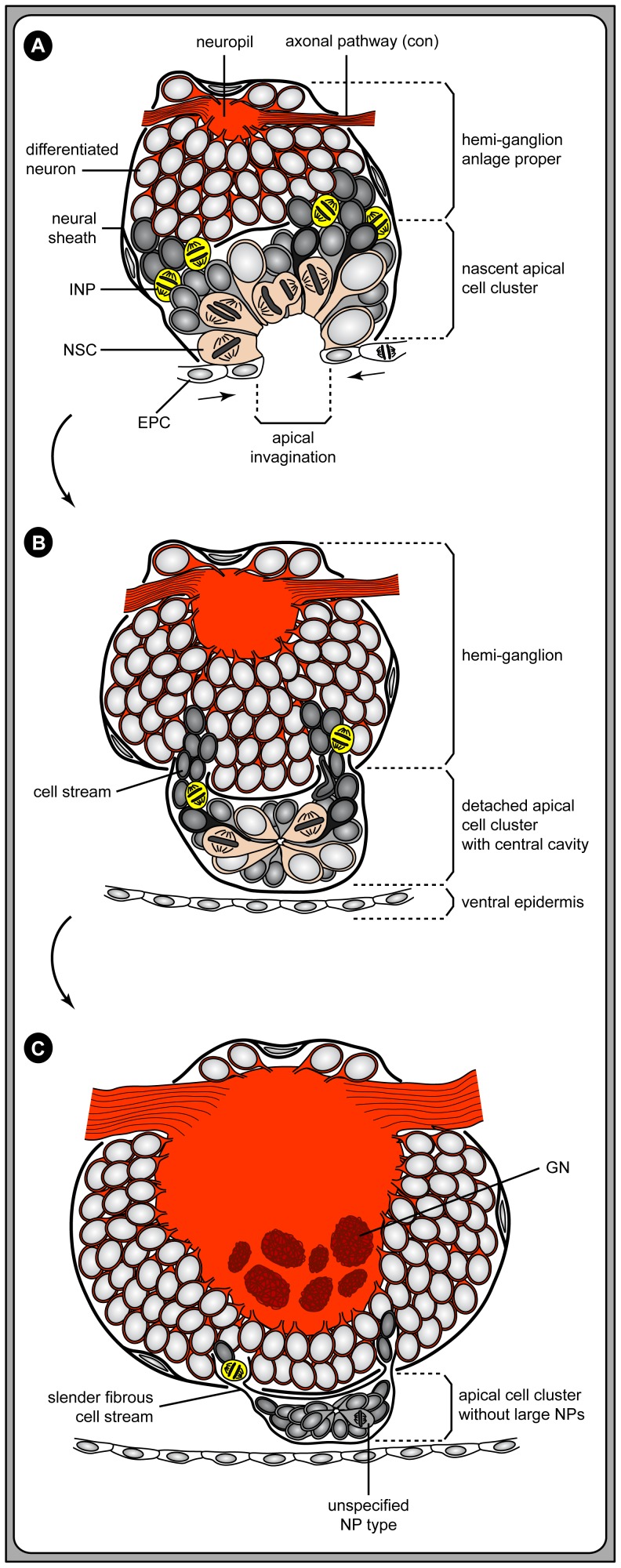
Formation of the apical CSSs during post-embryonic development of *Pseudopallene* sp. Schematic drawings in sagittal view. **A**: A deep apical invagination has formed in each hemi-segment, cells of the prospective epidermis (EPCs) covering its apical rim. Large spindle-shaped NSCs line the deep invagination and divide in asymmetrical fashion. Interspersed between the NSCs smaller flask-shaped cells are found, being in the process of basal immigration. NSCs and the interspersed cells form together the nascent apical cell cluster. Sub-apical to it, at least some of the immigrating cells divide, representing intermediate neural precursors (INPs). Characteristically, INPs are found in the still wide, nascent anterior and posterior cell streams that lead into the underlying hemi-ganglion anlage proper. **B**: All cells lining the deep central invagination detach from the ventral ectoderm, forming a fully separated apical cluster with a small central cavity at the ventral side of the hemi-ganglion. After the detachment process, the epidermis is apically closed. The cluster remains in contact with the underlying hemi-ganglion via well-defined anterior and a posterior cell streams. Along these streams, additional cell material migrates into the hemi-ganglion, being produced in the clusters as well as in the streams themselves. **C**: With ongoing development, the NSCs in the apical clusters decrease in size and become (at least morphologically) unidentifiable. The cell streams diminish to fibrous strands. Mitotic activity of (as yet uncharacterized) neural precursors persists in cluster and streams, but at a lower rate. The ganglionic neuropil increases considerably and ventrally glomerulus-like neuropils (GNs) are formed therein. Abbreviations: con  =  connective, EPC  =  epidermis cell, GN  =  glomerulus-like neuropil, INP  =  intermediate neural precursor, NP  =  neural precursor, NSC  =  neural stem cell.

Here, we have now shown that neurogenesis continues also during post-embryonic development of *Pseudopallene* sp. A considerable number of GCs is produced and incorporated into the segmental ganglion anlagen of the VNC, including those that have already been formed early on during embryonic development. This third phase of post-embryonic neurogenesis is characterized by the emergence of segmentally paired CSSs, which derive directly from the segmental invaginations of the VNE (Fig. 14A,B). The large NSCs remain identifiable in the apical cell clusters of early post-embryonic stages but do not seem to persist in later stages. Nonetheless, mitosis labeling reveals that cell divisions continue to occur within the clusters and along the cell streams, which penetrate the ganglionic neural sheath and connect the clusters to the underlying somata cortex (Fig. 14B,C). Based on all available evidence, we therefore clearly identify the apical CSSs as the sources for the additional GC material during the post-embryonic phase.

In line with our findings on *Pseudopallene* sp., the detachment of the invaginating VNE regions (‘ventral organs’) into the interior has been reported in all previous investigations on pycnogonids [82]–[84], [86], [87]. In further agreement with our results, a tiny cavity in the center of each detaching cell region, the initially high proliferation activity of the NSCs and their subsequent decrease in size and mitotic activity has been noted. In an attempt to clarify the possible function of the apical cell clusters, Dogiel [83], [84] cautiously proposed their connection to the slit glands of the ventral integument and the bifurcating sensory setae in *Nymphon spinosum*. Yet, we could clearly reject the existence of such a direct connection to the epidermal glandular and sensory system in *Pseudopallene* sp. Notably, Dogiel [83] also pointed out fibrous streams connecting the apical clusters of *N. spinosum* to the ganglionic somata cortex. Based on the structural similarities of apical clusters and connecting streams in *N. spinosum* and *Pseudopallene* sp., it seems very likely that identical cellular dynamics as described here can be recovered during post-embryonic nervous system development of nymphonids.

However, not in all investigated pycnogonid taxa, the detaching ‘ventral organs’ do subsequently form apical clusters located externally to the ganglion anlagen. In fact, apart from *Pseudopallene* sp., this feature has so far been solely described for nymphonid representatives (*N. spinosum* [83], [84]; *N. grossipes* [83], [84], [108]; *N. gracile* [85], [86]). In all other studied species, the large NSCs are instead incorporated into the ganglionic somata cortex, but remain nonetheless histologically distinguishable within the cortex's apical portion during the major part of post-embryonic development (*Callipallene brevirostris* and *Tanystylum orbiculare* [82]; *Anoplodactylus pygmaeus* and *A. petiolatus* [84]; *Achelia echinata*, *Anoplodactylus angulatus* and *Endeis spinosa* [86]; *Callipallene emaciata* [86], [87]; *Pycnogonum litorale* [unpubl. data]). Accordingly, the presence of spatially restricted cell proliferation systems giving rise to new GC material during late nervous system development is very likely to represent a general feature of late VNC development in pycnogonids. Yet, the anatomical separation of these neurogenic systems from the ganglia (in the sense of being located externally to the neural sheath instead of embedded in the somata cortex) is only encountered in some representatives. To all appearances, this anatomical separation is not even consistently recovered within Callipallenidae, as the development of different *Callipallene* species illustrates [82], [86], [87].

### The pycnogonid CSSs (advanced ‘ventral organs’) show similarities to the life-long neurogenic system of decapod crustaceans

We have identified the apical CSSs of the ventral ganglia of pycnogonids to be systems of post-embryonic neurogenesis. The apical cell cluster of each CSS – traditionally described as advanced ‘ventral organ’ stage – houses initially still large NSCs, which then gradually become morphologically untraceable. However, continuing cell proliferation shows that the CSSs contain still NPs after the ‘disappearance’ of the NSCs, although the exact NP type remains at present unclear (Fig. 14C). Based on our counts of ganglion cells as well as apical clusters cells and the observed restriction of cell proliferation to the CSSs, the apical clusters can be identified as neurogenic niches of post-embryonic development, i.e., they are cellular microenvironments that house NPs and act as sources for additional ganglion cell material. Since the CSSs persist throughout the entire post-embryonic development into adulthood, it seems even possible that *(i)* they have a life-long neurogenic function and *(ii)* house a NP type that possesses self-renewing properties. However, further studies are needed to clarify these two issues.

Interestingly, a series of studies performed on decapod crustaceans during the last two decades have identified a life-long neurogenic system, which shows several notable structural similarities to the pycnogonid CSSs. Already in histological studies [113]–[116], a so-called ‘deutocerebral organ’ had been identified at the ventral side of the brain of various decapods, but its function remained obscure. Using a combination of present-day techniques, this deutocerebral organ has now been unequivocally characterized as a system of life-long neurogenesis [59], [68], [69], [117]–[121] (see [64], [65] for reviews), with some contributions focusing on its post-embryonic development in crayfish [61], [122]. In all investigated decapods, this neurogenic system is exclusively affiliated with the deutocerebrum of the brain (in contrast to the segmental occurrence of CSSs in pycnogonids) and has been shown to give rise to cells that subsequently differentiate into local olfactory neurons and projection neurons [123]. These new-born local olfactory neurons and projection neurons contribute to the circuitries of the olfactory glomeruli in the olfactory lobe and of the glomeruli in the accessory lobe, respectively (both lobes being part of the deutocerebrum). The decapod neurogenic system almost always consists of a small cell cluster with a central cavity (the neurogenic niche), which is connected to the two deutocerebral somata clusters 9 and 10 (nomenclature following Sandeman et al. [124]) via two elongated cell streams (exception: separate niches in clusters 9 and 10 in spiny lobsters, e.g. [116], [119], [120]). Along the latter, cells migrate from the neurogenic niche into the clusters 9 and 10. Immunolabeling of PH3 and double-labeling experiments with *in-vivo* proliferation markers have demonstrated cell divisions in the niche itself and along the migratory streams as well as considerable proliferation in the region where the cell streams enter into clusters 9 and 10. Here, the produced immature cells finally differentiate into neurons. To date, it is still a matter of some debate what NP type is found in the niche (adult neural stem cells vs. non-self-renewing NPs recruited from outside the niche; see [68], [122] vs. [65], [67]; respectively).

Remarkably, the gross architecture of the CSSs in *Pseudopallene* sp. with its apical cell cluster containing a small central cavity and the two connecting cell streams, as well as the occurrence of cell proliferation in both sub-structures correspond to the decapod life-long neurogenic system. Furthermore, there are additional correspondences in the development of both systems. In crayfish, Sintoni et al. [61] have shown that the neurogenic niche and its connecting migratory streams are late developing structures, arising only in the course of post-embryonic development. They derive from the same region in which neural stem cells generate GC material during embryonic and early post-embryonic development. Both features are comparable to the CSS development in *Pseudopallene* sp. However, the niche of decapods has also been shown to be closely associated with the well developed circulatory system or even directly connected to it [61], [68], [69]. At least in crayfish, a growing body of evidence even suggests a replenishment of the NP pool of the niche via certain types of hemocytes [67]. Such a close structural interconnection is not as obvious in *Pseudopallene* sp., which lacks a complex circulatory system. In all pycnogonids, the circulatory system consists of a simple tube-like heart that pumps the hemolymph and is located dorsal to a horizontal septum (‘Dohrn's septum’), which merely divides the body cavity in a dorsal and a ventral sub-compartment [93], [125]. Nonetheless, the apical cell clusters of *Pseudopallene* sp. are still in direct contact to the hemolymph, being freely surrounded by it in the body cavity. Accordingly, interactions between hemocytes and the apical cell cluster would be possible.

### Spatial correlation of pycnogonid CSSs and glomerulus-like neuropils in the VNC

The structural similarities of the pycnogonid and the decapod systems as well as their corresponding neurogenic function raise the question whether even additional similarities at the level of the produced neural cell types might be encountered. The decapod life-long neurogenic systems are located in the deutocerebrum – the only region of the CNS that houses olfactory glomeruli as the primary centers of the olfactory pathway – and produce additional local and projection olfactory interneurons during adult life. Are there any indications for a similar role of the pycnogonid CSSs?

Unfortunately, the olfactory pathway of pycnogonids is only poorly understood. In fact, not even the exact position and structure of the first link in the chain – the pycnogonid chemoreceptors – is satisfactorily resolved. Stock [126] has proven in food preference investigations that pycnogonids do have a reasonably well developed chemical sense. Based on experiments involving the selective removal of chelifores, palps or ovigers, he could furthermore conclude that chemoreceptors are most likely distributed on different parts of the pycnogonid body including the walking legs, which was also assumed in other works [127], [128]. The hitherto most detailed description of the pycnogonid olfactory pathway was provided by Strausfeld et al. [129], [130]. They report the existence of glomerulus-like neuropils (GNs) in all walking leg ganglia of the pycnogonid *Ammothea hilgendorfi*, a neuroanatomical feature overlooked in all preceding histological studies [86], [87], [131]–[134]. These GNs are said to receive sensory input from setae on each walking leg and to be associated with olfactory interneurons that ascend into small mushroom body-like neuropils in the brain. However, only a rough scheme is depicted (see [129], their fig. 6, p. 22).

Here, we have identified – and for the first time shown – the GNs within the neuropil of the walking leg ganglia of *Pseudopallene* sp., confirming the findings of Strausfeld et al. [129], [130]. Furthermore, indications for the existence of small GNs in the sub-esophageal neuromeres of *Pseudopallene* provide an incentive for future investigations. The segmental occurrence of GNs within the pycnogonid VNC strongly speaks for the existence of olfactory interneurons in each of the ventral ganglia. In light of this, the segmental array of CSSs of *Pseudopallene* sp. at the ventral side of each VNC ganglion and the penetration of the cell streams into the ganglionic somata cortex close to the ventral GNs (Fig. 14C) represents a quite intriguing correlation. In correspondence to the decapod life-long neurogenic system, it might indicate an involvement of the pycnogonid CSS in the replenishment of interneurons associated with the primary olfactory centers. In order to test whether the pycnogonid CSSs are indeed involved in the production of olfactory neurons, future investigations on the post-embryonic and adult pycnogonid CSSs including *in-vivo* cell proliferation studies and dye-backfills (see e.g. [123]) from the ventral GNs are highly desirable.

### A late-developing (life-long) neurogenic system related to the central olfactory pathway as ancestral feature of the arthropod stem species?

Recent studies and reviews on life-long neurogenesis in decapods have predominantly highlighted similarities to vertebrate NSC niches, which among others also occur in association with the central olfactory pathway [65], [68], [135] (see [136] for neurogenic niches in vertebrates). Hence, the presence of life-long (olfactory) neurogenic systems has been concluded to represent a relatively common phenomenon especially in long-lived animals. Yet, the question of their homology, i.e., their derivation from a common origin, has been treated only cautiously (e.g. [65]).

Our identification of a late post-embryonic and potentially even life-long neurogenic system in pycnogonids with several striking similarities to the decapod system begs the question of a possible common origin of both systems either within the arthropod crown group, in the arthropod stem lineage, or even earlier during evolution. Given the very likely position of pycnogonids as sister group of the remaining chelicerates [2], [3], [78], [79] and the secure placement of decapods nested within the Tetraconata [1], [2], [5], [6], [137], an homology assumption would indicate such neurogenic systems to date back at least to the arthropod stem species. It can be further speculated that such neurogenic systems might have originally occurred in segmental array – similar to the pycnogonid CSSs – and as a consequence of lineage-specific specialization of segmental CNS units during arthropod evolution became restricted to those areas with a life-long turnover of neurons, such as the olfactory centers in the decapod deutocerebrum. Although appealing, this scenario lacks at the moment additional corroborating data. Future studies on pycnogonids are still indispensable in order to gain a better understanding of cell divisions dynamics, involved NP types and produced neural cell types in the pycnogonid CSSs. Only such additional studies can provide a sound basis for the critical evaluation of the similarities and differences between both systems and thereby enable well-founded homology assessments.

However, another indispensible step to test the plausibility of a common origin is the targeted search for the existence of corresponding neurogenic systems in the remaining arthropod lineages and – importantly – also in arthropod outgroups. Notably, an instance of a life-long (olfactory) neurogenic system may potentially be encountered in onychophorans as close relatives of arthropods [1], [3], [4], [78]. Ventrally attached to the adult onychophoran brain, the hypocerebral organ is found (e.g. [138], [139]), its function being at present still unsatisfactorily resolved. In the most recent contributions on this structure [139]–[141], it is considered whether it serves at least partially a glandular function. Remarkably, however, several hypocerebral organ features are similar to the decapod life-long neurogenic system and (where known) to the pycnogonid CSSs:

*(i)* The hypocerebral organ is a direct derivative of the protocerebral neuroectoderm, which thickens and invaginates during development – forming also a so-called ‘ventral organ’ – and eventually detaches interiorly [138], [139], [142]. The same relationships hold for the pycnogonid CSSs and also the sub-structures of the crayfish neurogenic system have their origin in the same neuroectodermal region as the deutocerebral neuroblasts earlier on [61]. *(ii)* The adult hypocerebral organ is an apical cell cluster attached ventrally to the brain, the cluster cells being arranged around a central cavity with inwards directed apical poles and peripherally displaced nuclei [139], [140]. *(iii)* It is not innervated by the brain but connected to it via cell streams [140]. *(iv)* Numerous mitoses have been observed among the hypocerebral organ cells during development and in adults [140]. *(v)* The hypocerebral organ cells are characterized by numerous mitochondria, a well-developed Golgi apparatus, conspicuous cytoplasmic vesicles and the existence of tight cell junctions towards the central cavity [140], similar to the predominant type I cells of the crayfish neurogenic niche [69]. *(vi)* Although so-called ‘ventral organs’ develop along the entire length of the onychophoran trunk, only the protocerebral region is characterized by the interior detachment and persistence of the hypocerebral organ. In contrast to all extant arthropods, onychophorans possess a fully developed limb pair affiliated with the protocerebral region, the ‘primary antennae’ [143] (nomenclature following Scholtz and Edgecombe [144]). Receiving input from this sensory appendage, onychophoran olfactory glomeruli are found exclusively in the protocerebral neuropil together with the mushroom bodies [145], [146]. Hence, hypocerebral organ and the primary and secondary centers of the central olfactory pathway show a ‘fitting’ segmental affiliation that might further support our hypothesis.

Based on this list of features, we consider the onychophoran hypocerebral organ a promising candidate for more detailed investigations that seek to shed light on a potential neurogenic function. As in pycnogonids, experiments applying cell proliferation markers with focus on the adult nervous system coupled, for instance, with dye-backfills or subsequent immunolabeling of a range of neurotransmitters would help to test this notion [123]. However, even if the homology between the onychophoran hypocerebral organs, the pycnogonid CSSs and the deutocerebral niche in decapod crustaceans is plausible, the differences in the segmental affiliations (protocerebrum, walking leg segments, deutocerebrum) of these niche-structures pose a problem for character polarization and our understanding of their evolution.

## Supporting Information

Movie S1 **Cell count in walking leg ganglion 1 and affiliated apical cluster of PS 6 of** ***Pseudopallene*** **sp.** Acetylated tubulin (red) labeling with Hoechst (blue) counterstain, Imaris volume (blend) and oblique slicer. The movie shows the ventral two thirds of the walking leg ganglion and starts in ventral view. The ganglion rotates 360 degrees around the z-axis (counter-clockwise). Each nucleus of the morphological left hemi-ganglion and apical cell cluster has been marked with a spot (red and light red, respectively). To illustrate the principle of the cell count, an oblique slicer passes from ventral to dorsal to show the single virtual section planes in which nuclei were manually marked.
(AVI)

Movie S2 **Nascent apical cell clusters and cell divisions in walking leg ganglion 1 of early PS 1 of** ***Pseudopallene*** **sp.** Acetylated tubulin (red) and PH3 (yellow) labeling with Hoechst (blue) counterstain, virtual transverse section series, Imaris oblique slicer passing from posterior to anterior through the ganglion anlage. Note the deep paired apical pits as well as numerous divisions of NSCs and several sub-apical INPs. While the nascent apical cell clusters remain connected to the underlying ganglion proper via diffuse anterior and posterior cell streams, a distinct separation of both regions is apparent in the middle of the section series. Dorsally, flattened nuclei of glial cells are scattered in the ganglionic neural sheath.
(AVI)

Movie S3 **Detaching apical cell clusters and cell divisions in walking leg ganglion 1 of late PS 1 of** ***Pseudopallene*** **sp.** Tyrosine tubulin (red) and PH3 (yellow) labeling with Hoechst (blue) counterstain, virtual transverse section series, Imaris oblique slicer passing from posterior to anterior through the ganglion anlage. The apical pits have completely closed and a small central cavity is formed in the resulting apical cell cluster. The formerly apical poles of the cluster cells (including NSCs) are directed towards the cavity. A thin layer formed by epidermal cells with small apico-basally flattened nucleus covers the cell clusters. Note persisting size differences between the cluster cells, NSC divisions within clusters as well as some smaller INP divisions in the anterior and posterior cell streams. Several intensely labeled pycnotic bodies point to the occurrence of cell death between apical clusters and ganglion proper and in part also within the cell streams.
(AVI)

Movie S4 **Completely detached apical cluster-stream-systems in walking leg ganglion 1 of early PS 2 of** ***Pseudopallene*** **sp.** Acetylated tubulin (red) and PH3 (yellow) labeling with Hoechst (blue) counterstain, virtual transverse section series, Imaris oblique slicer passing from posterior to anterior through the ganglion. The apical cell clusters are separated from the ventral epidermis by longitudinal muscles. The cell streams are more defined, having decreased in diameter. Size differences between cluster cells are not as prominent any longer, but in part still discernible. Morphological identification of NSCs has become ambiguous. Only a single mitosis is PH3-labeled in the left CSS. Note some pycnotic bodies in the cell streams and within the somata cortex. The ganglionic neuropil has significantly gained in volume.
(AVI)

Movie S5 **Ovigeral CSSs in the composite sub-esophageal ganglion of PS 3 of** ***Pseudopallene*** **sp.** Acetylated tubulin (red) and PH3 (yellow) labeling with Hoechst (blue) counterstain, virtual transverse section series, Imaris oblique slicer passing from posterior to anterior through the ganglion. Distinct size differences between cluster cells are no longer assessable. The connecting cell streams have further diminished to fibrous strands along which only few cells with elongated nucleus appear to immigrate into the somata cortex. One PH3-labeled cell in the left anterior cell stream is in found prophase. On the right side, the slicer proceeds into the more antero-lateral palpal CSS. Note several flattened nuclei of glial cells in the neural sheath. Antero-dorsally, the ganglion is slightly damaged.
(AVI)
